# UDP-glucose pyrophosphorylase as a target for regulating carbon flux distribution and antioxidant capacity in *Phaeodactylum tricornutum*

**DOI:** 10.1038/s42003-023-05096-3

**Published:** 2023-07-19

**Authors:** Ruihao Zhang, Baohua Zhu, Changze Sun, Yun Li, Guanpin Yang, Yan Zhao, Kehou Pan

**Affiliations:** 1 Key Laboratory of Mariculture (Ocean University of China), Ministry of Education, Qingdao, 266003 China; 2 Function Laboratory for Marine Fisheries Science and Food Production Processes, Qingdao National Laboratory for Marine Science and Technology, Qingdao, 266100 China; 3 College of Marine Life Sciences, Ocean University of China, Qingdao, 266003 China

**Keywords:** Metabolic engineering, Molecular engineering in plants

## Abstract

UDP-glucose pyrophosphorylase (UGPase) is a key enzyme for polysaccharide synthesis, and its role in plants and bacteria is well established; however, its functions in unicellular microalgae remain ill-defined. Here, we perform bioinformatics, subcellular localization as well as in vitro and in vivo analyses to elucidate the functions of two UGPs (UGP1 and UGP2) in the model microalga *Phaeodactylum tricornutum*. Despite differences in amino acid sequence, substrate specificity, and subcellular localization between UGP1 and UGP2, both enzymes can efficiently increase the production of chrysolaminarin (Chrl) or lipids by regulating carbon flux distribution without impairing growth and photosynthesis in transgenic strains. Productivity evaluation indicate that UGP1 play a bigger role in regulating Chrl and lipid production than UGP2. In addition, UGP1 enhance antioxidant capacity, whereas UGP2 is involved in sulfoquinovosyldiacylglycerol (SQDG) synthesis in *P. tricornutum*. Taken together, the present results suggest that ideal microalgal strains can be developed for the industrial production of Chrl or lipids and lay the foundation for the development of methods to improve oxidative stress tolerance in diatoms.

## Abstract (web summary)

Both UDP-glucose pyrophorylases (UGP) UGP1 and UGP2 in microalgae are involved in lipid production and storage molecule regulation but differ in the type of lipids produced and effect on antioxidant capacity.

## Introduction

Increasingly serious problems such as climate change, depletion of fossil resources, and the food crisis have prompted efforts to develop environmentally friendly and sustainable photosynthetic platforms for the production of valuable products such as biofuels and bio-based chemicals^1^. Microalgae are photoautotrophic eukaryotic organisms that can use carbon fixed by photosynthesis to synthesize multiple carbon storage compounds such as lipids, polysaccharides, and pigments, simultaneously achieving carbon reduction and green production^2–5^.

The development of microalgae-based cell factories has emerged as an environmentally sustainable solution^6^. However, the accumulation of intracellular products in microalgae is often accompanied by the slowdown of cell growth, which affects the synthesis of microalgal products^7^. In addition, microalgae are exposed to various environmental sources of oxidative stress (high temperature, high light, nutrient deficiencies) during large-scale cultivation outdoors, and these environmental stresses cause reactive oxygen species (ROS) accumulation, which severely affects the growth of algal cells, and thus, decreases productivity^8,9^. Therefore, an economically viable microalgae industry requires optimization of productivity in the context of a sharp increase in demand for high-value products^6^. The biosynthesis of carbon storage products in microalgae is associated with competition for the same carbon precursors^10,11^; adjusting the distribution of photosynthetically fixed carbon could thus be an effective way to boost the synthesis of target products^3^.

*Phaeodactylum tricornutum*, a typical diatom, has been developed as a designable and scalable photosynthetic cell factory because of its rapid growth rate, mature genetic transformation technology, and abundant high-value compounds^6^. Two major carbon storage compounds in *P. tricornutum*, chrysolaminarin (Chrl) and lipids, are highly valuable marketed products that have broad application prospects in aquaculture, pharmaceuticals, and bioenergy^5,12^. Chrl is the main carbohydrate energy reserve of diatoms and has attracted research interest because of its antioxidant and antitumor effects^13,14^. However, little is known about the factors regulating Chrl biosynthesis, and the biosynthetic pathway of Chrl has not been fully elucidated^15,16^.

Lipid synthesis in *P. tricornutum* has been studied extensively. Stress induction and genetic engineering can effectively increase lipid production^17^. However, lipid accumulation caused by environmental stress is usually accompanied by reduced biomass, which is not suitable for industrial production^18–20^. Genetic engineering to increase lipid production focuses mainly on the regulation of enzymes in the lipid synthesis pathway, whereas the regulation of key nodes of other metabolic pathways to improve lipid synthesis has received little attention^21,22^. The biosynthesis of carbohydrates such as Chrl competes with lipid synthesis for the photosynthetic carbon source^3,14,23^. Thus, redirecting the carbon flux toward lipid synthesis by regulating key nodes in the Chrl synthesis pathway may be an effective strategy to increase lipid accumulation.

UDP-glucose (UDPG) pyrophosphorylase (UGPase) catalyzes the synthesis of UDPG from Glc-1-P and UTP^24^. UDPG is a key substrate or precursor for the synthesis of sucrose and polysaccharides in plants, and UGPase thus plays a central role in carbohydrate metabolism and plant development^25^. UGPase plays an important role in regulating carbon flux distribution in higher plants. Coleman et al. overexpressed *UGPase* in hybrid poplars, thereby altering the distribution of carbon flux in starch and cellulose^26^. Wang et al. overexpressed cotton *UGPase* in *Arabidopsis thaliana*, and the results showed that UGPase plays a key role in carbon flux distribution, especially in cellulose synthesis^27^. It is speculated that there are two UGPs (UGP1 and UGP2) in *P. tricornutum*^28,29^. A preliminary study of UGP1 from our group showed that UGP1 was associated with the synthesis of Chrl and lipid^16^, but the related study on UGP2 has not been reported.

In this study, we identified and characterized UGP1 and UGP2 in *P. tricornutum* and determined the substrate specificity, phylogenetic relationships, and subcellular localization of the two enzymes. The roles of UGP1 and UGP2 in carbon flux distribution were demonstrated by gene overexpression and RNA interference, which provided regulatory targets for enhancing Chrl and lipid accumulation in diatoms by genetic engineering. Transcriptome analysis and biochemical evidence indicated that UGPase decreases ROS production and cell mortality by regulating the expression of genes related to ROS metabolism and programmed cell death (PCD), suggesting a strategy to improve oxidative stress tolerance in microalgae.

## Results

### Characterization of UGP1 and UGP2 sequences

**Fig. 1:**
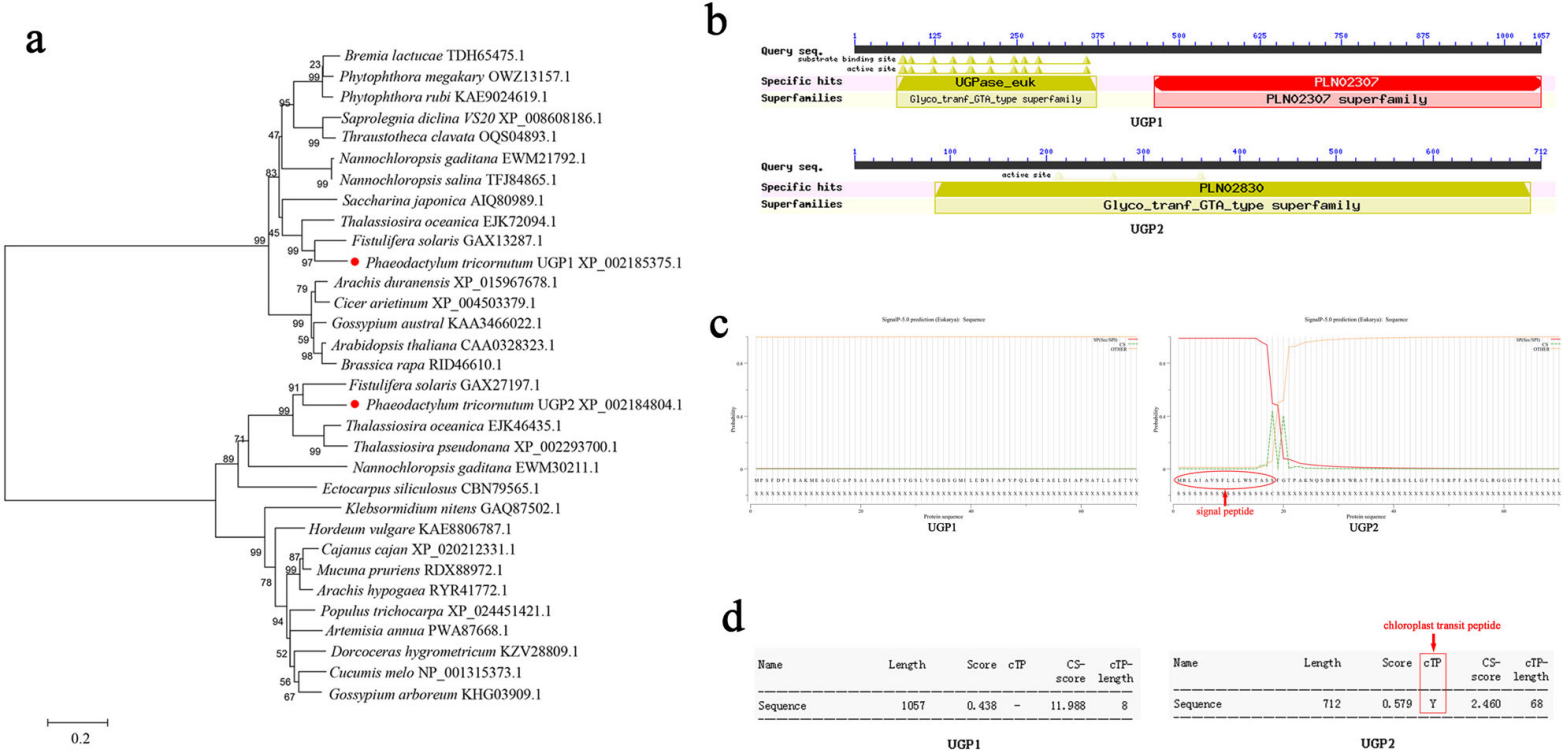
Characterization of UGP1 and UGP2 sequences. **a** Phylogenetic tree based on amino acid sequences of UGPs by neighbor-joining method. The red dots highlight UGP1 and UGP2. **b** Prediction of conserved domains in UGP1 and UGP2. **c** Prediction of signal peptide in UGP1 and UGP2 by using web-based prediction tools SignalP. The red arrow indicates that UGP2 has signal peptide. **d** Prediction of chloroplast transit peptide in UGP1 and UGP2 by using web-based prediction tools ChloroP. The red arrow indicates that UGP2 has chloroplast transit peptide. UGP1 and UGP2, UDP-glucose pyrophosphorylase.

To analyze the evolutionary relationship between UGP1 and UGP2, the amino acid sequences of UGPs from various species were retrieved, and a phylogenetic tree was constructed using the NJ method. As shown in Fig. 1a, both UGP1 and UGP2 shared the highest sequence similarity and homology with the diatom *Fistulifera solaris*. However, they could not be clustered into one group in the phylogenetic tree, indicating low sequence homology between them. Conserved domain prediction showed that UGP1 had the typical conserved structural domains of glycosyltransferases and phosphoglucomutases, whereas UGP2 had only the conserved structural domain of glycosyltransferases (Fig. 1b). The subcellular localization of UGP1 (without the signal peptide) was predicted to be in the cytoplasm and UGP2 (containing the signal peptide and chloroplast transit peptide) was predicted to be in the chloroplast using the online tools SignalP and ChloroP (Fig. 1c, d). In summary, although UGP1 and UGP2 shared the same structural domain and might catalyze the same reaction, their low sequence similarity and different subcellular localization suggest that they play different roles in *P. tricornutum*.

### Detection of UGP1 and UGP2 subcellular localization

**Fig. 2:**
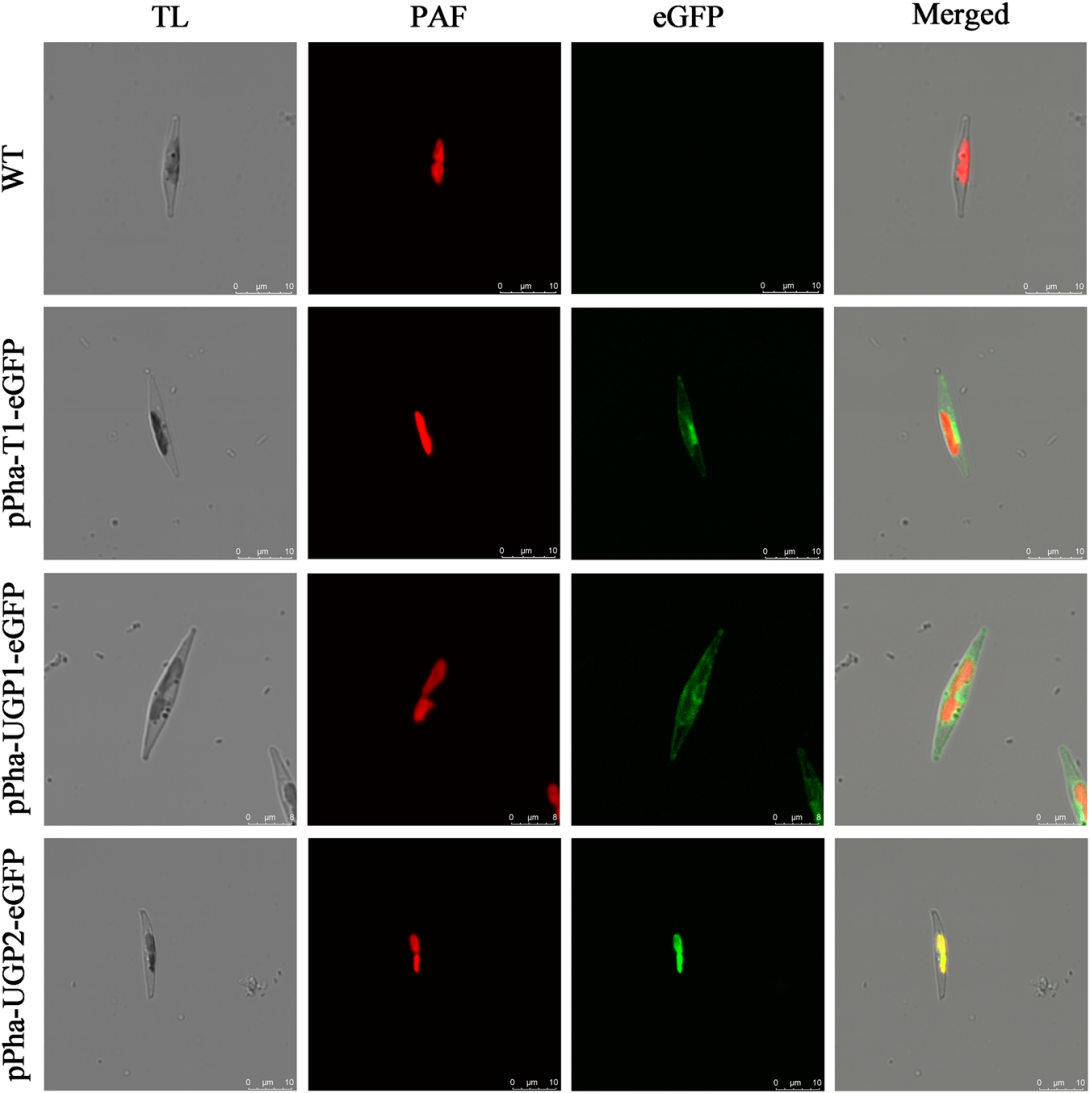
Subcellular localization of UGP1 and UGP2. WT wild-type *Phaeodactylum tricornutum*; pPha-T1-eGFP: transformation of eGFP alone; pPha-UGP1-eGFP: transformation of UGP1 fused to eGFP; pPha-UGP2-eGFP: transformation of UGP2 fused to eGFP; TL transmitted light; PAF plastid autofluorescence; eGFP enhanced green fluorescent protein; Merged: overlay of plastid and green fluorescence. UGP1 and UGP2, UDP-glucose pyrophosphorylase. Scale bars in WT, pPha-T1-eGFP and pPha-UGP2-eGFP represents 10 μm; Scale bars in pPha-UGP1-eGFP represents 8 μm.

To further study the precise subcellular localization of UGP1 and UGP2, we constructed fusion expression vectors of UGP1 and UGP2 with enhanced green fluorescent protein (eGFP), respectively (Supplementary Fig. 1). The results were shown in Fig. 2, the attachment of UGP1 to eGFP resulted in the green fluorescence signals located in the cytoplasm, the attachment of UGP2 to eGFP caused the green fluorescence signals to be located in the chloroplast. These results confirmed that UGP1 and UGP2 were localized in the cytoplasm and chloroplast, respectively, which were consistent with the results predicted in Fig. 1.

### Determination of substrate specificities of UGP1 and UGP2 using in vitro enzyme assays

Kinetic parameters were determined using purified biologically active UGP1 and UGP2 recombinant proteins (Supplementary Fig. 2). The initial velocity of the enzymatic reaction against varying concentrations of sugar-1-P (or a sugar-1-P analogue) was fitted to the Michaelis-Menten equation to determine K_m_, K_cat_, and K_cat_/K_m_ values with fixed saturating concentration of UTP. As shown in Table 1, in the presence of four different substrates (Glc-1-P/Gal-1-P/Xyl-1-P/GlcA-1-P), UGP1 had the highest affinity and catalytic activity against Glc-1-P. The K_cat_/K_m_ value against Glc-1-P was 27.83-fold and 40.97-fold higher than that against the two other substrates, Gal-1-P and GlcA-1-P. However, UGP1 did not exhibit catalytic activity for Xyl-1-P. Unlike UGP1, UGP2 exhibited catalytic activity for all four substrates. UGP2 showed the highest affinity and catalytic activity for Glc-1-P, with a more than 100-fold higher K_cat_/K_m_ value than those measured for the other three substrates, Gal-1-P, Xyl-1-P, and GlcA-1-P. In addition, the affinity and catalytic efficiency of UGP2 for Glc-1-P were significantly higher than those of UGP1, with a K_cat_/K_m_ value 2.83-fold higher than that of UGP1. These data indicate that UGP1 and UGP2 have different specificities towards sugar-1-P (or a sugar-1-P analogue). Although the optimal substrate for both enzymes is Glc-1-P, UGP2 showed a stronger ability to catalyze the conversion of Glc-1-P than UGP1.

**Table 1:** Kinetic parameters of recombinant UGP1 and UGP2 by the substrate of sugar-1-P (or a sugar-1-P analogue).

	UGP1			UGP2		
	K_m_ (mM)	K_cat_ (s^-1^)	K_cat_/K_m_ (s^-1^ μM^-1^)	K_m_ (mM)	K_cat_ (s^-1^)	K_cat_/K_m_ (s^-1^ μM^-1^)
Glc-1-P	0.04 ± 0.002^a^	2355.03 ± 11.14^c^	57.08 ± 2.10^b^	0.01 ± 0.002^a^	2783.13 ± 50.34^b^	218.51 ± 23.17 ^b^
Gal-1-P	0.40 ± 0.013^b^	785.88 ± 5.25^b^	1.98 ± 0.05^a^	0.41 ± 0.009^b^	641.20 ± 5.13^a^	1.58 ± 0.05^a^
GlcA-1-P	0.49 ± 0.030^c^	663.51 ± 13.53^a^	1.36 ± 0.07^a^	0.38 ± 0.006^b^	641.49 ± 6.94^a^	1.68 ± 0.02^a^
Xyl-1-P	ND	ND	ND	1.01 ± 0.025^c^	612.25 ± 1.24^a^	0.61 ± 0.02^a^

Experiments were carried out in triplicate at 37˚C in 100 mM Hepes buffer (pH 7.5) by varying the concentration of sugar-1-P (or a sugar-1-P analogue) substrate with fixed saturating concentrations of UTP (1 mM). UGP1 and UGP2, UDP-glucose pyrophosphorylase. ND, no detectable activity was observed in this experiment. *n*  =  3 biologically independent samples. The data were shown as the mean ± standard deviation (SD). Values with different letters (a, b, c,) in the same column indicate a significant difference between them (*p* < 0.05).

### Molecular characterization of transgenic microalgae

**Fig. 3:**
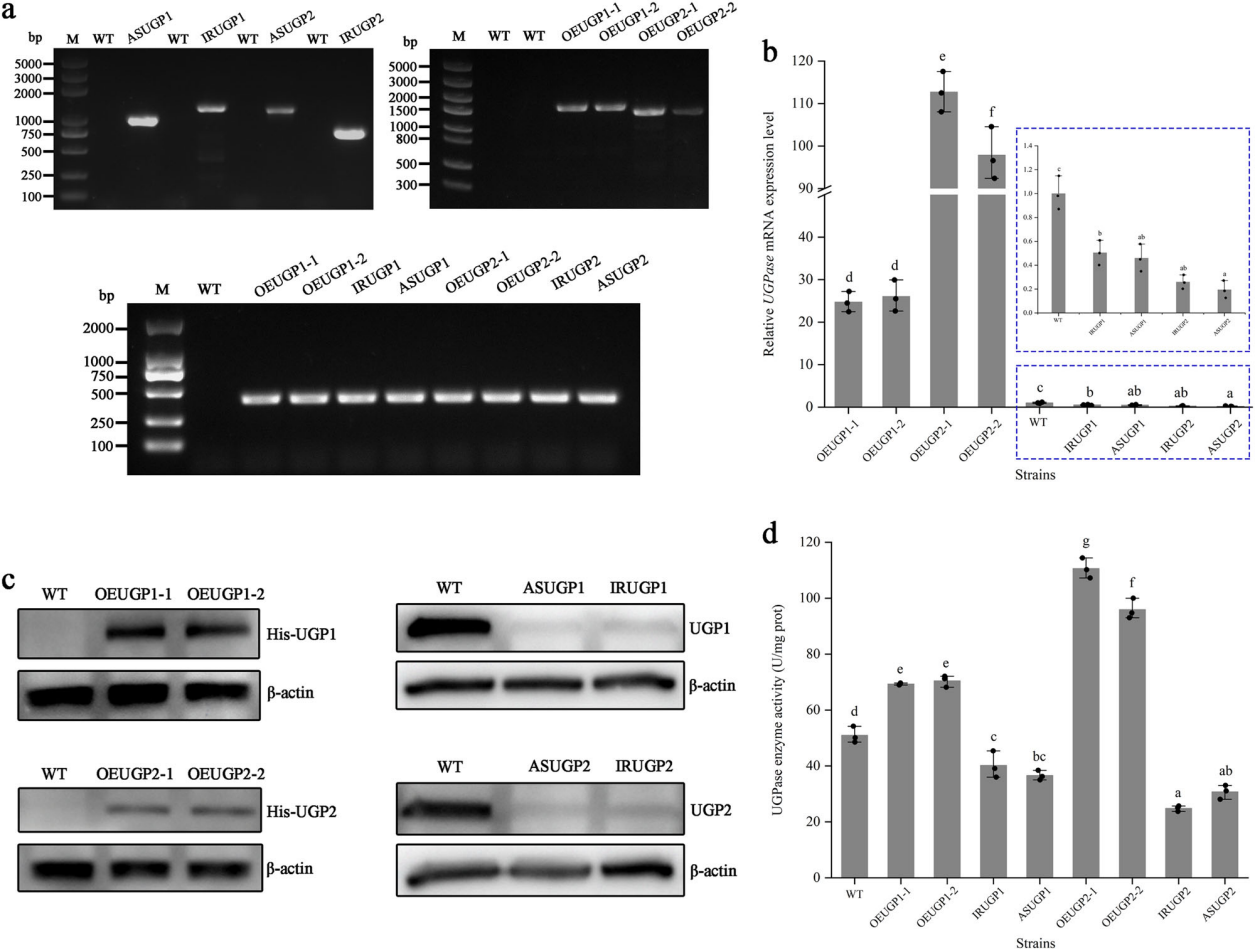
Validation of transgenic strains. **a** Preliminary molecular analysis of transgenic strains. The integration of expression vector in the resistant clones was analyzed by PCR. M, 2- and 5-kb DNA size marker. **b** Relative transcript levels of *UGP1* and *UGP2* determined by qPCR. *UGP1* and *UGP2* transcript abundance was quantified with WT in normal cultivation as the standard and normalized to endogenous histone H4 gene. **c** Western blot analysis of overexpression strains using an anti-His antibody. Western blot analysis of silenced strains using anti-UGP1 and anti-UGP2 antibodies. β-actin was used as an internal control. **d** UGPase activity in transgenic strains and WT. UGP1 and UGP2, UDP-glucose pyrophosphorylase. WT represents Wild-type strain; ASUGP1 and IRUGP1 represent *UGP1* silenced strains; OEUGP1-1 and OEUGP1-2 represent *UGP1*-overexpressing strains; ASUGP2 and IRUGP2 represent *UGP2* silenced strains; OEUGP2-1 and OEUGP2-2 represent *UGP2*-overexpressing strains. *n* = 3 biologically independent samples. The data were shown as the mean ± standard deviation (SD). Differences among groups were determined by one-way ANOVA and Tukey’s test. Values with different letters (a, b, c, d, e, f, g) indicate a significant difference between them (*p* < 0.05). The blue dashed boxes highlight the relative transcript levels of *UGP1* and *UGP2* in WT and silenced strains.

The integration of the expression vector into the *P. tricornutum* genome was assessed by PCR using specific primers (Supplementary Table 1). As shown in Fig. 3a, the amplified fragments of *sh ble* and target genes (*UGP1* and *UGP2*) connected to *sh ble* were detected in the resistant clones but not in the WT. These results suggest that the recombinant vectors carrying the target fragments were successfully integrated into the genome of *P. tricornutum*.

To verify the expression levels of *UGP1* and *UGP2*, quantitative real-time PCR (qPCR) analysis was performed in transgenic strains. The transcript abundance of *UGP1* and *UGP2* was significantly higher in overexpression strains and lower in silenced strains than in the WT strain (Fig. 3b). These results indicated that overexpression and silencing vectors integrated into the *P. tricornutum* genome were successfully transcribed. Furthermore, overexpression and silencing of *UGP1* did not change the expression level of *UGP2*, and overexpression and silencing of *UGP2* did not change the expression level of *UGP1*, indicating that there was no genetic complementarity between *UGP1* and *UGP2* (Supplementary Fig. 4).

The protein levels in overexpression lines were analyzed by western blotting using an anti-His antibody. Cross-reacting protein bands with molecular weights of 115.4- and 78.5-kDa were observed in UGP1- and UGP2-overexpressing strains, respectively, whereas such cross-reactive bands were not detected in the WT (Fig. 3c). This suggests that the overexpression vectors were integrated into the *P. tricornutum* genome and successfully expressed His-tagged UGP1 and UGP2 fusion proteins. The protein levels in silenced lines were analyzed by western blotting using anti-UGP1 and anti-UGP2 antibodies, respectively. As shown in Fig. 3c, the protein levels of UGP1 and UGP2 were significantly lower in silenced strains than in the WT strain, indicating that silencing vectors successfully inhibited the synthesis of UGP1 and UGP2 proteins.

UGPase activity was also significantly altered in transgenic versus WT strains. As shown in Fig. 3d, overexpression of both *UGP1* or *UGP2* significantly increased the enzymatic activity of UGPase, and silencing *UGP1* or *UGP2* significantly reduced the enzyme activity of UGPase.

### Growth and photosynthetic performance of transgenic microalgae

**Fig. 4:**
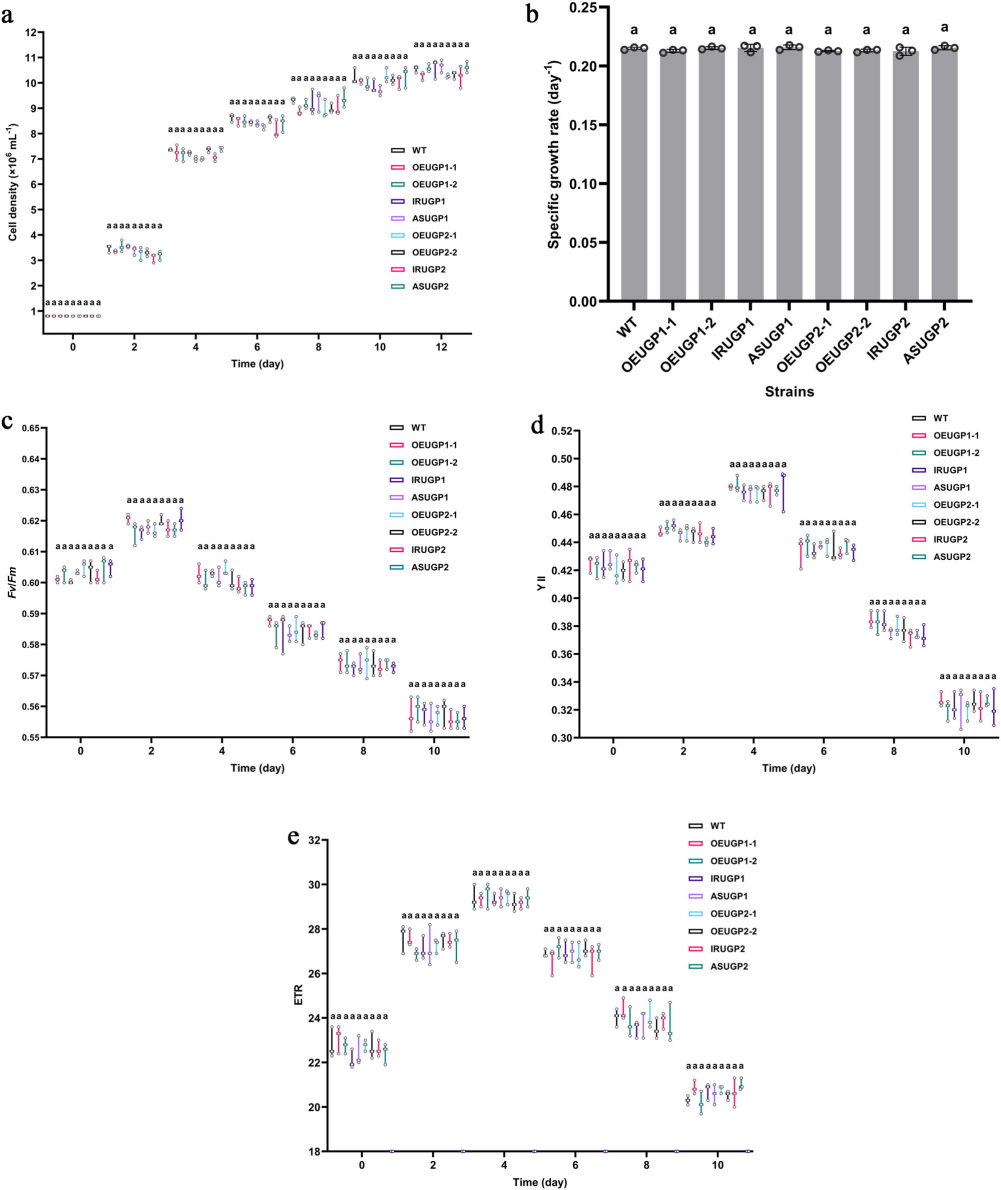
Growth and photosynthetic performance of WT and transgenic strains. **a** Growth curves. **b** Specific growth rate. **c** The maximum quantum yield of PSII (*Fv*/*Fm*). **d** The effective quantum yield (YII). **e** The electron transport rate (ETR). UGP1 and UGP2, UDP-glucose pyrophosphorylase. WT represents Wild-type strain; ASUGP1 and IRUGP1 represent *UGP1* silenced strains; OEUGP1-1 and OEUGP1-2 represent *UGP1*-overexpressing strains; ASUGP2 and IRUGP2 represent *UGP2* silenced strains; OEUGP2-1 and OEUGP2-2 represent *UGP2*-overexpressing strains. *n* = 3 biologically independent samples. The data were shown as the mean ± standard deviation (SD). Differences among groups were determined by one-way ANOVA and Tukey’s test. Values with same letters (a) indicate no significant difference between them (*p* > 0.05).

As shown in Fig. 4a, b, the WT and all transgenic strains showed similar growth curves during culture and their cell densities and specific growth rates were not significantly different. In addition, chlorophyll fluorescence parameters such as *Fv*/*Fm*, YII, and ETR did not differ significantly between transgenic and WT strains during culture (Fig. 4c–e). These results suggest that overexpression and silencing of *UGP1* and *UGP2* did not affect the growth and photosynthetic activity of microalgae.

### UGP1 and UGP2 are key regulators of the Chrl biosynthetic pathway

**Fig. 5:**
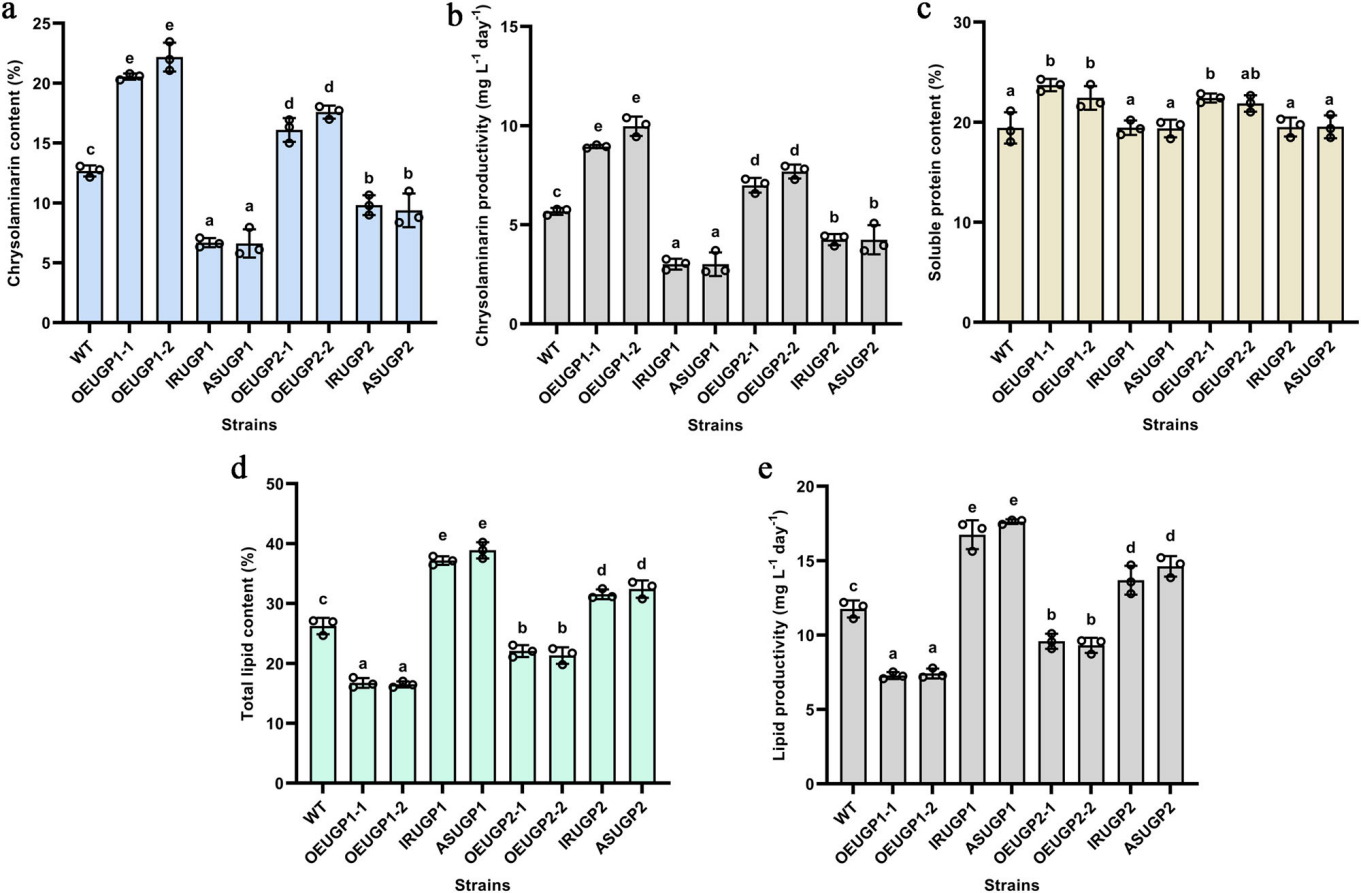
Analysis of primary metabolites of WT and transgenic strains. **a** The chrysolaminarin content. **b** Chrysolaminarin productivity on the 12th day. **c** The soluble protein content. **d** The total lipid content. **e** Lipid productivity on the 12th day. UGP1 and UGP2, UDP-glucose pyrophosphorylase. WT represents Wild-type strain; ASUGP1 and IRUGP1 represent *UGP1* silenced strains; OEUGP1-1 and OEUGP1-2 represent *UGP1*-overexpressing strains; ASUGP2 and IRUGP2 represent *UGP2* silenced strains; OEUGP2-1 and OEUGP2-2 represent *UGP2*-overexpressing strains. *n* = 3 biologically independent samples. The data were shown as the mean ± standard deviation (SD). Differences among groups were determined by one-way ANOVA and Tukey’s test. Values with different letters (a, b, c, d, e, f) indicate a significant difference between them (*p* < 0.05).

The Chrl content was 75.01% higher in *UGP1*-overexpressing strains and 38.81% higher in *UGP2*-overexpressing strains than in the WT, whereas the Chrl content was significantly lower in all silenced strains (Fig. 5a). These results indicate that UPG1 and UGP2 are key regulators of the Chrl biosynthetic pathway, and overexpression of *UGP1* and *UGP2* is an effective way to promote Chrl accumulation.

The Chrl content of *UGP1*-overexpressing strains was significantly higher than that of *UGP2*-overexpressing strains, whereas the UGPase activity of *UGP1*-overexpressing strains was lower than that of *UGP2*-overexpressing strains (Figs. 3d, 5a). Similarly, among the silenced strains, *UGP2* silenced strains with lower UGPase activity had a higher Chrl content than *UGP1* silenced strains with higher UGPase activity (Figs. 3d, 5a). These results suggest that the role of UGP1 in the production of Chrl is more important than that of UGP2, and UGP2 may have other functions that need to be studied.

To further evaluate the application potential of UGP1 and UGP2 in Chrl production, the synthesis of Chrl was examined in WT and transgenic strains. As shown in Fig. 5b, Chrl production was higher in *UGP1*-overexpressing strains than in *UGP2*-overexpressing strains, indicating that *UGP1* is a more promising regulatory target for the production of Chrl.

### UGP1 and UGP2 modulate lipid biosynthesis by regulating carbon flux distribution

As shown in Fig. 5c, the soluble protein content of the silenced strains did not differ significantly from that of the WT, and only the protein content of the overexpression strains was slightly increased. However, modulating the expression of *UGP1* and *UGP2* had a marked effect on lipid synthesis (Fig. 5d). The lipid content of all silenced strains was significantly higher than that of the WT. The lipid content of the two *UGP1* silenced strains increased by 41.60% and 48.15%, which was a greater increase than that of *UGP2* silenced strains. The lipid content of *UGP1* and *UGP2* overexpression strains was significantly lower than that of the WT. These findings indicate that UGP1 and UGP2 negatively regulate lipid synthesis in *P. tricornutum*, and UGP1 plays a greater role in modulating lipid accumulation than UGP2. Correlation analysis showed that the lipid content in transgenic strains was significantly and negatively correlated with the content of Chrl. These results suggest that carbon flux shifted from the sugar synthesis pathway to the lipid synthesis pathway, thus boosting lipid accumulation.

The application potential of UGP1 and UGP2 in lipid production was evaluated by comparing lipid production between WT and transgenic strains. As shown in Fig. 5e, silenced strains produced significantly higher amounts of lipid than WT strains, especially *UGP1* silenced strains. These results suggest that silencing the expression of *UGP1* or *UGP2* to shift the carbon flux towards the lipid synthesis pathway could be a strategy to enhance lipid production, and *UGP1* is a more effective target to promote lipid accumulation.

### The role of UGP2 in SQDG biosynthesis in *P. tricornutum*

**Fig. 6:**
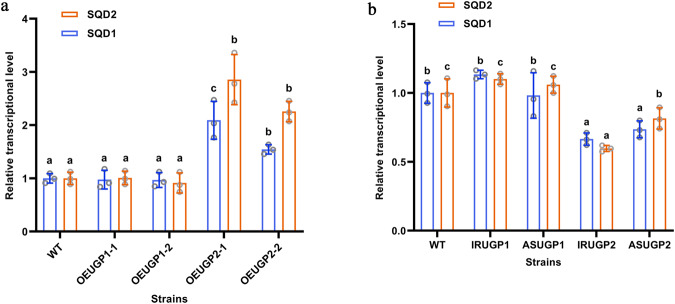
Transcript abundance analysis of *SQD1* and *SQD2* in WT and transgenic strains. **a** Relative transcript level of the overexpressed strains *SQD1* and *SQD2* by qPCR and normalized by histone H4 gene. **b** Relative transcript level of the silenced strains *SQD1* and *SQD2* by qPCR and normalized by histone H4 gene. UGP1 and UGP2, UDP-glucose pyrophosphorylase. *SQD1*, UDP-sulfoquinovose synthase gene; *SQD2*, sulfolipid synthase gene. WT represents Wild-type strain; ASUGP1 and IRUGP1 represent *UGP1* silenced strains; OEUGP1-1 and OEUGP1-2 represent *UGP1*-overexpressing strains; ASUGP2 and IRUGP2 represent *UGP2* silenced strains; OEUGP2-1 and OEUGP2-2 represent *UGP2*-overexpressing strains. *n* = 3 biologically independent samples. The data were shown as the mean ± standard deviation (SD). Differences among groups were determined by one-way ANOVA and Tukey’s test. Values with different letters (a, b, c) indicate a significant difference between them (*p* < 0.05).

The results of a previous study^30^ together with the present results suggested that UGP2 plays an important role in the synthesis of SQDG in *P. tricornutum*. The role of UGP2 in SQDG synthesis was thus analyzed by detecting the transcription of two key genes in the SQDG synthesis pathway, *SQD1* and *SQD2*, and their correlation with the transcript levels of *UGP1* and *UGP2* was analyzed. As shown in Fig. 6, compared with the WT, the transcription levels of *SQD1* and *SQD2* in *UGP2-*overexpressing strains were significantly increased by up to 1.09- and 1.87-fold, respectively. The transcription levels of *SQD1* and *SQD2* in *UGP2* silenced strains were significantly decreased by up to 33.50% and 40.30%, respectively, compared with that in the WT. Correlation analysis confirmed that the transcription levels of *SQD1* and *SQD2* were significantly and positively correlated with those of *UGP2* (Supplementary Table 3). However, the transcription levels of *SQD1* and *SQD2* did not differ significantly between *UGP1* transgenic strains and the WT strain. These results indicate that UGP2 plays a vital role in the biosynthesis of SQDG by regulating the expression of *SQD1* and *SQD2* in *P. tricornutum*. The content of SQDG should be determined in future research in *UGP2* transgenic strains.

### Overexpression of *UGP1* increases the antioxidant capacity of *P. tricornutum*

**Fig. 7:**
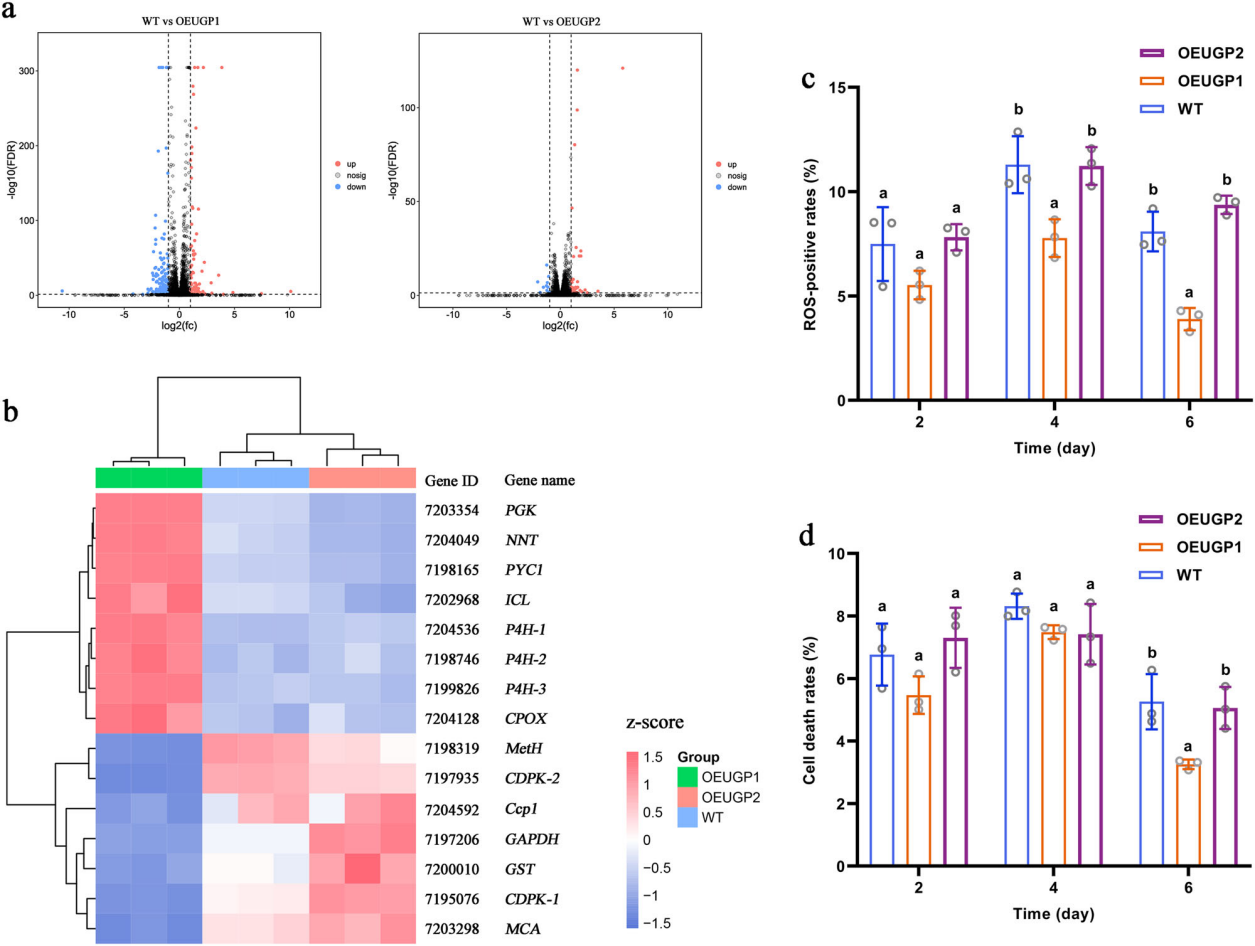
Transcriptome and biochemical analysis of overexpressed and WT strains. **a** Volcano plot showing the number of up- and down-regulated differentially expressed genes (DEGs) (FDR < 0.05, |log_2_fold change | ≥ 1) in each comparison group. **b** Heatmap illustrating the expression levels of DEGs related to antioxidant and cell death in WT and overexpression strains. **c** Changes of ROS-positive rates in overexpressed and WT strains during culture. **d** Changes of cell death rates in overexpressed and WT strains during culture. UGP1 and UGP2, UDP-glucose pyrophosphorylase. WT represents Wild-type strain; OEUGP1 represents *UGP1*-overexpressing strains; OEUGP2 represents *UGP2*-overexpressing strains. *n* = 3 biologically independent samples. The data were shown as the mean ± standard deviation (SD). Differences among groups were determined by one-way ANOVA and Tukey’s test. Values with different letters (a, b) indicate a significant difference between them (*p* < 0.05). *PGK*, phosphoglycerate kinase gene; *NNT*, NAD(P)^+^ transhydrogenase gene^;^ *PYC1*, pyruvate carboxylase gene; *ICL*, isocitrate lyase gene; *P4H*, prolyl 4-hydroxylase gene; *CPOX*, coproporphyrinogen III oxidase gene; *MetH*, methionine synthase gene; *CDPK*, calcium dependent protein kinase gene; *Ccp1*, cytochrome c peroxidase gene; *GAPDH*, glyceraldehyde-3-phosphate dehydrogenase gene; *GST*, glutathione S-transferase gene; *MCA*, metacaspase gene. Gene ID represents the number of the genes in the National Center of Biotechnology Information (NCBI) database.

To further explore other functions of UGP1 and UGP2 in *P. tricornutum*, we performed RNA-seq transcriptome analysis of WT and overexpression lines (Fig. 7a, b, and Supplementary Table 2). We detected 257 DEGs between WT and *UGP1-*overexpressing strains (FDR < 0.05, |log_2_fold change | ≥ 1), of which 82 genes were upregulated and 175 genes were downregulated in *UGP1*-overexpressing strains compared with the WT. By contrast, only 59 DEGs were identified between WT and *UGP2-*overexpressing strains (FDR < 0.05, |log_2_fold change | ≥ 1), including 47 upregulated genes and 12 downregulated genes. Further analysis of the differential genes revealed that overexpression of *UGP1* significantly changed the expression levels of genes related to ROS metabolism and PCD, whereas overexpression of *UGP2* did not change the expression levels of these genes. Among them, marked increases were observed in transcripts encoding coproporphyrinogen III oxidase and NAD(P)^+^ transhydrogenase, which play essential roles in removing ROS and maintaining the antioxidant capacity^31,32^. Transcripts of the metacaspase gene, a key enzyme that positively regulates PCD in microalgae^33^, were significantly reduced. The increased ROS scavenging capacity resulted in significantly decreased transcript levels of other enzymes related to ROS scavenging, such as glutathione S-transferase, cytochrome c peroxidase, glyceraldehyde-3-phosphate dehydrogenase, and methionine synthase. Meanwhile, the expression levels of genes associated with sensing and responding to hypoxic conditions were significantly increased due to enhanced ROS scavenging capacity. Among them, the transcripts of prolyl 4-hydroxylase, a key regulator of hypoxia-inducible factor-1, were significantly increased. The transcripts of phosphoglycerate kinase and isocitrate lyase, which are key genes of the glycolysis pathway and glyoxylate cycle that maintain the energy supply and fundamental metabolism under hypoxic conditions^34,35^, were significantly increased.

To determine whether the altered expression levels of genes associated with oxidative stress and PCD resulted in physiological changes, we measured ROS production and cell death rates in WT and overexpression strains during cultivation (Fig. 7c, d). ROS production and cell death rates were significantly lower in *UGP1*-overexpressing strains than in WT strains, showing a 51.98% reduction in ROS production rates and a 38.02% reduction in cell death rate on the 6th day of cultivation. However, ROS production and cell death rates did not differ significantly between WT and *UGP2*-overexpressing strains throughout the experimental period. These results suggest that overexpression of *UGP1* decreases intracellular ROS levels by modulating the expression of related genes, thereby improving the antioxidant capacity and survival of microalgae.

### Validation of RNA-seq data by qPCR analysis

**Fig. 8:**
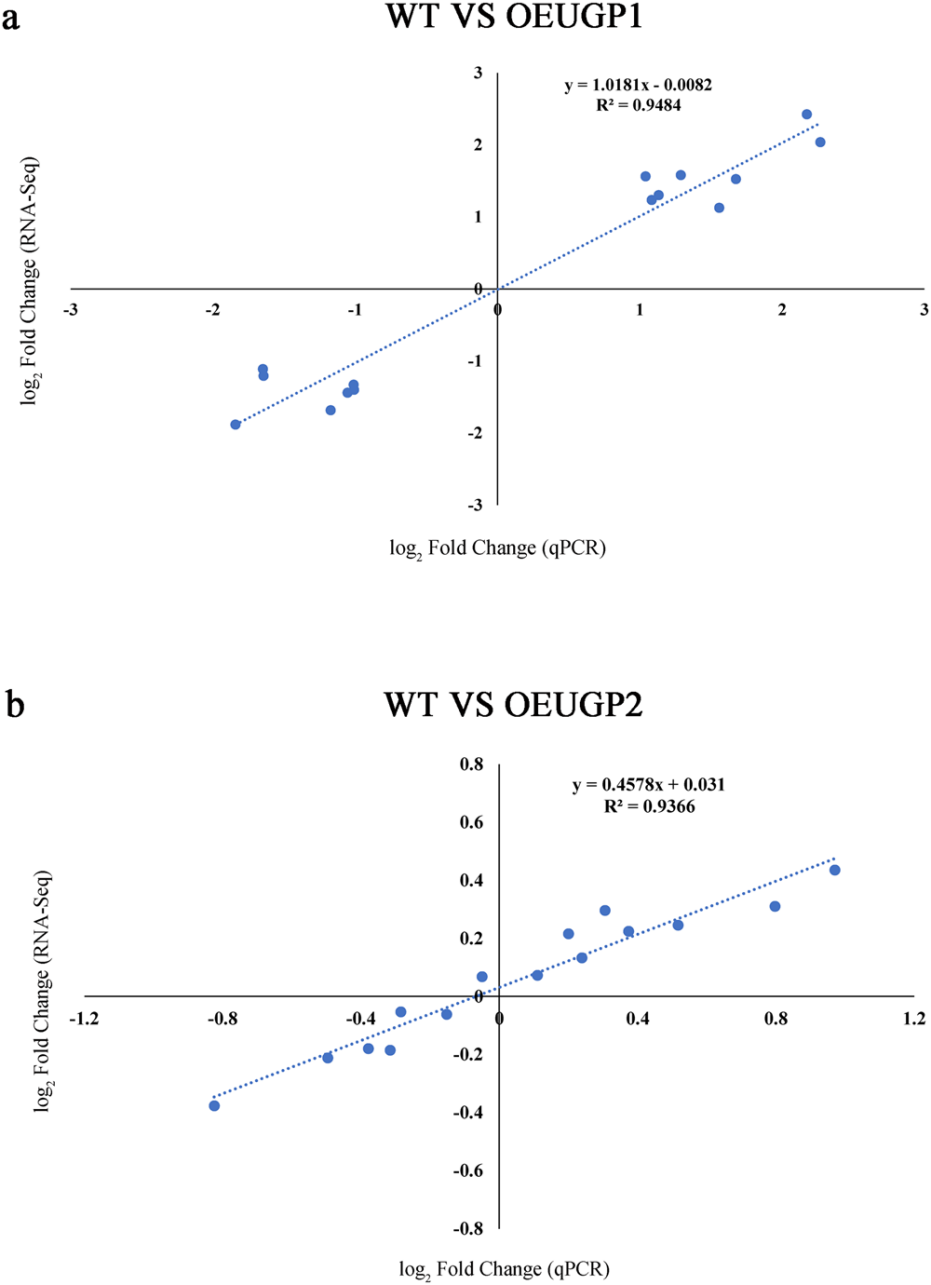
Correlation analysis between transcriptome and qPCR data. **a** Correlation analysis of WT and OEUGP1 comparison group. **b** Correlation analysis of WT and OEUGP2 comparison group. UGP1 and UGP2, UDP-glucose pyrophosphorylase. WT represents Wild-type strain; OEUGP1 represents *UGP1*-overexpressing strains; OEUGP2 represents *UGP2*-overexpressing strains.

To verify the reliability of the RNA-Seq data, we selected 15 DEGs of Fig. 7b for qPCR analysis. The results showed that the qPCR data exhibited significant correlation with the RNA-Seq data (Fig. 8), indicating the reliability of the RNA-Seq data.

## Discussion

The two main carbon storage products in *P. tricornutum*, Chrl and lipids, have promising applications in pharmaceuticals, cosmetics, and bioenergy^5^. Chrl biosynthesis competes with lipid biosynthesis for photosynthetically fixed carbon^23,36^. Therefore, regulating the photosynthetic carbon flux may be an effective strategy to modulate the accumulation of target products. UGPase, a key enzyme in plant polysaccharide biosynthesis, plays a key role in regulating carbon allocation in higher plants^26,27^. However, comprehensive studies of its role in carbon flux distribution are lacking, particularly in microalgae.

In this study, two UGPs (UGP1 and UGP2) were identified and characterized in *P. tricornutum*. Although UGP1 and UGP2 share the same catalytic function (catalytic production of UDPG), phylogenetic tree analysis revealed that they have low amino acid sequence homology, which is inconsistent with research results in higher plants. Sowokinos et al. and Meng et al. reported that UGPases have highly conserved amino acid sequences, with more than 90% identity among isoforms^24,37^. Regarding substrate specificity and catalytic efficiency, previous studies reported that UGP1 has strict specificity towards Glc-1-P, whereas UGP2 can use different sugar-1-P forms (or sugar-1-P analogues) as substrates, and the affinity of UGP1 for Glc-1-P is greater than that of UGP2 in higher plants^24,38^. However, in the present study, we found that both UGP1 and UGP2 could catalyze the conversion of various sugar-1-P forms (or sugar-1-P analogues), and UGP2 has broader substrate specificity and higher catalytic efficiency than UGP1 in *P. tricornutum*.

Key amino acids such as Cys and Lys in UGPase play a crucial role in catalytic activity and substrate binding in higher plants^39,40^. Kleczkowski et al. reported that differences in amino acid sequence can affect the three-dimensional structure or fold of proteins, thereby affecting protein function^38^. Therefore, differences in substrate specificity and reaction efficiency between UGP1 and UGP2 may depend on differences in amino acid sequence. Furthermore, the functional diversity of different enzymes and isozymes involved in UDPG synthesis in plants is affected by subcellular localization^41^. Our study demonstrated for the first time experimentally that UGP1 was localized in the cytoplasm, while UGP2 was localized in the chloroplast. Therefore, the differences in substrate specificity and catalytic efficiency between UGP1 and UGP2 observed in this study may be attributed to differences in subcellular localization.

Photosynthetically fixed carbon can be allocated to multiple pathways for the synthesis of major macromolecules such as carbohydrates, lipids, and proteins^42^. Methods have been developed for promoting the accumulation of target products by regulating the carbon flux distribution in microalgae. In *Chlamydomonas reinhardtii*, knockdown of the CrPEPC1 gene redirects the carbon flux from sugar synthesis to lipid synthesis^43^. Similarly, downregulation of the Chrl synthase gene redirects the carbon flux toward lipid biosynthesis in the diatom *T. pseudonana*^44^. Yang et al. reported that overexpression of the phosphoglucomutase gene significantly increases the Chrl content and decreases the lipid content in *P. tricornutum*^14^. Similar results were obtained in the present study, suggesting that *UGP1* and *UGP2* are good candidate genes for regulating carbon flux distribution between Chrl and lipids to promote the accumulation of target products.

Biomass is a key factor in the commercial production of microalgal products^14^. However, accumulation of high-value products in microalgae is detrimental to cell growth, which is a potential limitation to microalgal-based production^7^. Studies on microorganisms have demonstrated that this is attributed to competition in carbon metabolic flow between cell growth and product synthesis^45,46^. Therefore, the balance of carbon flux between cell growth and target product synthesis must be considered when selecting regulatory targets. Li et al. reported that the lipid content of a *Chlamydomonas starchless* mutant defective in *AGPase* is significantly increased, whereas the growth rate is reduced by 12.3%^36^. Radakovits et al. also observed decreased cell growth rates in transgenic strains^47^. These results do not promote the production of microalgal products. We found that accumulation of Chrl or lipids in transgenic strains did not negatively affect cell growth and photosynthesis, suggesting that regulating the expression of *UGP1* and *UGP2* could improve production and accumulation of microalgal products.

Transcriptome analysis and biochemical evidence suggested that overexpression of *UGP1* could improve antioxidant capacity and reduce cell mortality by regulating the expression of genes related to ROS metabolism and cell death, and this is the first report of this effect in *P. tricornutum*. Microalgae are exposed to various environmental sources of oxidative stress during outdoor large-scale cultivation, and these environmental stresses can inhibit algal growth^8,9^. However, the mechanism underlying oxidative stress responses in microalgae remains unclear. The present experimental results support the role of UGP1 in enhancing antioxidant capacity and provide ideas for the study of oxidative stress responses in microalgae. Xia et al. demonstrated that Chrl has strong antioxidant activity^13^. However, in the present study, although the Chrl content of *UGP2*-overexpressing strains was significantly increased, there were no changes in antioxidant-related genes and phenotypes, indicating that the improved antioxidant capacity of *P. tricornutum* was not necessarily related to Chrl. The mechanism underlying the role of UGP1 in regulating the antioxidant capacity of microalgae remains unclear and needs further study.

Although UGP2 was not involved in regulating the antioxidant capacity of microalgae, we found that it might be involved in the biosynthesis of SQDG. SQDG is a sulfur-containing lipid in plants, and thus provides the bulk of the structural lipids in photosynthetic membranes^30^. Although the two key enzymes in SQDG biosynthesis, SQD1 and SQD2, have been characterized at the genetic and enzymatic levels, little is known about the UDPG supply mechanism in the SQDG biosynthesis pathway in microalgae. In this study, we showed that the transcription levels of *SQD1* and *SQD2* were significantly and positively correlated with those of *UGP2*. Combined with the subcellular localization and catalytic properties of UGP2, we speculated that UGP2 plays an essential role in SQDG biosynthesis by providing UDPG in *P. tricornutum*. These findings need to be further confirmed by quantifying the SQDG content.

**Fig. 9:**
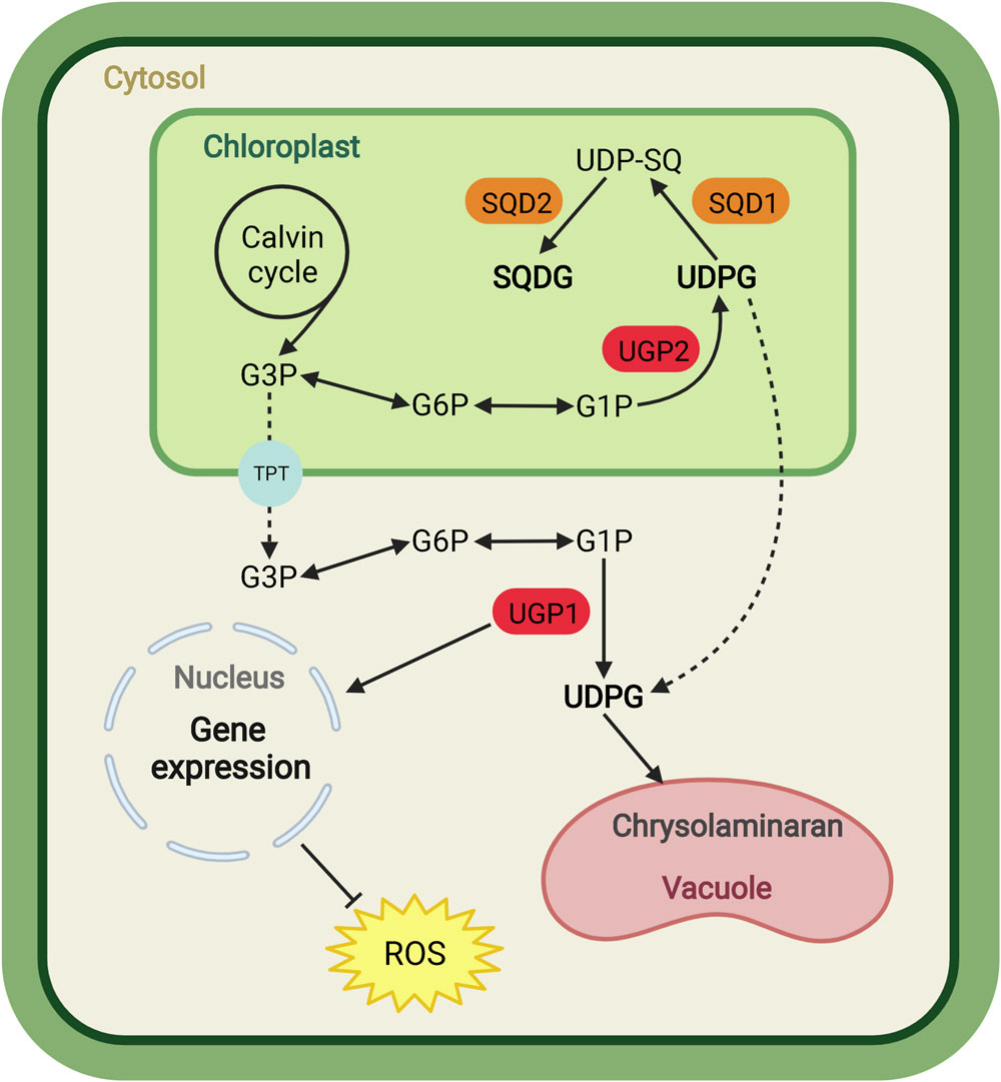
A working model for UGP1 and UGP2 functions in *P. tricornutum*. UGP1 located in the cytosol participates in Chrl biosynthesis and regulates the antioxidant capacity of algal cells. UGP2 located in the chloroplast participates in Chrl and SQDG biosynthesis. G3P glyceraldehyde 3-phosphate, G6P glucose-6-phosphate, G1P glucose-1-phosphate, TPT triose phosphate translocator, UDPG uridine diphosphate glucose, UDP-SQ UDP-sulfoquinovose, SQDG sulfoquinovosyldiacylglycerol, SQD1 UDP-sulfoquinovose synthase, SQD2 sulfolipid synthase, UGP1 and UGP2 UDP-glucose pyrophosphorylase, ROS reactive oxygen species. Red highlights UGP1 and UGP2; orange highlights SQD1 and SQD2; yellow highlights ROS.

In summary, two UGPs were identified and characterized in this study, and the critical role of UGP1 and UGP2 in synthesizing Chrl and regulating carbon flux distribution was confirmed (Fig. 9). The results suggest that regulating the expression of *UGP1* and *UGP2* could modulate the production of Chrl and lipids in microalgae without impairing algal growth and photosynthesis, thus providing a direction to improve the efficacy of microalgal-based production. In addition, our work identified functions of UGP1 and UGP2 in enhancing the antioxidant capacity of microalgae and in SQDG synthesis, respectively (Fig. 9), suggesting directions for the in-depth study of oxidative stress response mechanisms and the SQDG biosynthetic pathway in microalgae.

## Materials and methods

### Strains and growth conditions

Wild-type (WT) *P. tricornutum* Bohlin (Laboratory of Applied Microalgae Biology, Ocean University of China) and transgenic strains were grown in sterile seawater f/2 medium at 21 ± 1 °C under 50 µmol photons·m^-2^·s^-1^ provided by white fluorescent tubes with a 12 h:12 h light/dark cycle. To eliminate the effects of Zeocin^TM^ (Invitrogen, Carlsbad, CA, USA) on microalgae, transformants were cultured in Zeocin^TM^-free f/2 medium for more than three successive subculture cycles prior to experimental measurements.

### UGP1 and UGP2 sequence analysis and bioinformatic predictions

The amino acid sequences of UGP1 [50444] and UGP2 [23639] were retrieved from the JGI *P. tricornutum* database based on the descriptions of Huang et al.^28^. and Kroth et al.^29^. Amino acid sequence similarity between species was detected using BLAST on NCBI (https://blast.ncbi.nlm.nih.gov/Blast.cgi), and phylogenetic trees were constructed using the amino acid sequences of UGP proteins from various species using the neighbor-joining (NJ) method in MEGA 5. Conserved domains of UGP1 and UGP2 were predicted using NCBI’s conserved domain database^48^. The subcellular localization of UGP1 and UGP2 was predicted using the web-based prediction tools SignalP and ChloroP^49^.

### Subcellular localization of UGP1 and UGP2 in *P. tricornutum*

To construct fusion vectors of *UGP1* and *UGP2* with *eGFP*, *eGFP* was connected to the 3’-end of the *UGP1* and *UGP2*, and then inserted into pPha-T1 vector. The pPha-T1 vector with only *eGFP* inserted was used as a positive control vector. The complete vector maps constructed were shown in Supplementary Fig. 1. The eGFP, chloroplast autofluorescence and the superposition signal of the two fluorescence were observed by laser scanning confocal microscopy (Leica, Germany). The excitation and emission wavelengths of eGFP were 488 nm and 520–540 nm, respectively. The excitation and emission wavelengths of chloroplast autofluorescence were 488 nm and 630–700 nm, respectively.

### In vitro UGP1 and UGP2 enzyme assay

Expression and purification of recombinant UGP1 and UGP2 proteins. The target fragments for the construction of the expression vector were amplified using specific primers (Supplementary Table 1) and inserted into the pEASY-Blunt E2 expression vector to obtain the recombinant plasmids pEASY-UGP1 and pEASY-UGP2. The recombinant plasmids were transformed into *E. coli* BL21 (DE3). To induce the production of recombinant proteins, bacterial cells were first cultivated at 37 °C until the OD_600_ value reached 0.6. Isoprpyl β-D-thiogalactopyranoside (IPTG) was then added at a concentration of 1 mM, and cells were grown for an additional 20 h at 18 °C. The cells were collected by centrifugation at 4 °C and resuspended in lysis buffer. The recombinant proteins were extracted and purified by His Trap Ni-NTA (GE Healthcare, USA) affinity chromatography. The purity of recombinant proteins was assessed by SDS-PAGE. The concentration of recombinant proteins was determined by the BCA method (Solarbio, Beijing, China).

UGP1 and UGP2 enzyme assay. The enzymatic activities of UGP1 and UGP2 recombinant proteins were measured using the Plant UDPase Assay Kit (Genmed Scientifics Inc., Shanghai, China). The reaction system was constructed according to the manufacturer’s instructions, and UGPase activity was measured by monitoring absorbance changes at 30 min intervals at 340 nm using a UV-3310 spectrophotometer (Hitachi). One unit of UGPase activity was defined as 1 nmol of NADPH generated by per mg of protein per minute. Kinetic constants of UGP1 and UGP2 proteins were determined as described by Meng et al.^24^. and Hao et al.^22^. Briefly, approximately 6 ng of purified protein was added to a 1 mL assay mixture composed of 100 mM Hepes (pH 7.5), 5 mM MgCl_2_, 1 mM UTP, and 0.08-1.6 mM Glc-1-P/Gal-1-P/Xyl-1-P/GlcA-1-P. After mixing thoroughly and incubating at 37 °C for 20 min, the mixture was heated at 100 °C for 5 min to terminate the reaction. The PPi produced was hydrolyzed to Pi by reacting with 0.5 units of inorganic pyrophosphatase (Sigma). Pi was quantified using Malachite Green Phosphate Detection Kit (Beyotime, Shanghai, China) according to the manufacturer’s instructions. Finally, the initial velocities at different substrate concentrations were determined by calculating the PPi content and fitted to the Michaelis-Menten equation to determine the kinetic parameters K_m_, K_cat_, and K_cat_/K_m_ using Origin software for different substrates.

### Overexpression and silencing vectors construction and microalgal transformation

*UGP1* and *UGP2* (GenBank accession: XM_002185339.1 and XM_002184768.1) were amplified by polymerase chain reaction (PCR) using cDNA from *P. tricornutum* as the template. Fragments required for the construction of overexpression and silencing vectors were amplified from *UGP1* and *UGP2*, respectively. To construct the overexpression vectors, the full-length coding sequences of *UGP1* and *UGP2* were amplified by PCR using specific primers containing the Xbal I/Hind III sites and 6x His tag sequence (see Supplementary Table 1), then inserted into the pPha-T1 plasmid to generate pPha-T1-OEUGP1 and pPha-T1-OEUGP2, respectively. Additionally, antisense and inverted-repeat vectors were constructed for gene silencing. For the antisense vector construction, antisense fragments of *UGP1* and *UGP2* were amplified using the primers UGP1-As-f (containing the XbaI site), UGP1-AS-r (containing the EcoRI site), UGP2-As-f (containing the XbaI site) and UGP2-As-r (containing the KpnI site) (Supplementary Table 1). The two fragments were inserted into pPha-T1 in antisense orientation to generate the pPha-T1-ASUGP1 and pPha-T1-ASUGP2 vectors. For construction of inverted-repeat vectors, the longer fragments UGP1-IR1 and UGP2-IR1 were amplified with the primers UGP1-IR1-f (containing the EcoRI site), UGP1-IR1-r (containing the XbaI site), UGP2-IR1-f (containing the Hind III site), and UGP2-IR1-r (containing the XbaI site), and the shorter fragments UGP1-IR2 and UGP2-IR2 were amplified with the primers UGP1-IR2-f (containing the Hind III site), UGP1-IR2-r (containing the XbaI site), UGP2-IR2-f (containing the KpnI site), and UGP2-IR2-r (containing the XbaI site) (Supplementary Table 1). UGP1-IR1 and UGP1-IR2 were inserted into pPha-T1 in the forward and reverse directions to form the inverted-repeat vectors pPha-T1-IRUGP1 and pPha-T1-IRUGP2. The vector maps constructed in this section were shown in Supplementary Fig. 3. All vectors were constructed with a bleomycin-resistant gene (*sh ble*) cassette.

The recombinant plasmids constructed were transformed into *P. tricornutum* as described by Zaslavskaia et al.^50^. Briefly, the constructed plasmids were wrapped using tungsten particles according to the manufacturer’s protocol, and the tungsten particles were bombarded into the algal cells by using a Bio-Rad Biolistic PDS-1000/He Particle Delivery System (Bio-Rad Laboratories, Hercules, CA, USA). The bombarded cells were placed in a lighted incubator (21 ± 1 °C, 50 µmol photons·m^-2^·s^-1^, 12 h:12 h light/dark cycle) for 48 h recovery culture. Then, algal cells were resuspended in 1 mL of sterile seawater f/2 medium and plated onto f/2 solid medium (containing 1% agar) supplemented with 100 µg mL^-1^ Zeocin^TM^ to select transgenic strains.

### Molecular identification of transformants

To detect the integration of the expression vectors in transgenic strains, PCR analysis was performed with the specific primers and genomic DNA as the template. The primers used in this section are listed in Supplementary Table 1.

The expression levels of *UGP1* and *UGP2* in transgenic strains were determined by measuring the relative abundance of *UGP1* and *UGP2* transcripts at the exponential stage by qPCR. Briefly, RNA was extracted from WT and transformants using the Total RNA Kit I (Omega Bio-Tek, USA), and the relative expression levels of *UGP1* and *UGP2* were quantified using the Applied Biosystems QuantStudio5 Real-Time PCR System with Fast SYBR Green Master Mix. The histone H4 gene was used as the internal control^51^. The relative transcript abundance was calculated using the 2^−ΔΔCt^ method^52^. Primers used for qPCR are shown in Supplementary Table 1. The annealing temperature of *UGP1*, *UGP2* and *H4* primers was 60 °C, and their amplification efficiencies were 97.011, 103.863 and 96.304, respectively.

The expression levels of the UGP1 and UGP2 proteins in transgenic strains were determined by western blot analysis. The anti-His antibody was used to detect the UGP1 and UGP2 recombinant proteins carrying a His-tag at the C-terminus in overexpression strains. The expression levels of UGP1 and UGP2 proteins in silenced strains were detected by anti-UGP1 and anti-UGP2 antibodies. Total proteins were extracted from WT and transgenic strains for western blot analysis. Anti-His antibody (dilution of 1:1000; Cell Signaling Technology), anti-UGP1antibody (dilution of 1:5000; Genecreate Biological Engineering, Wuhan, China.) and anti-UGP2 antibody (dilution of 1:5000; Genecreate Biological Engineering, Wuhan, China.) were used as the primary antibodies, and β-actin (dilution of 1:5000; Cell Signaling Technology) was used as the internal control.

### Measurement of UGPase activity

UGPase enzyme activities of WT and transgenic strains were measured using the Plant UDPase Assay Kit (Genmed Scientifics Inc., Shanghai, China) according to the experimental procedure described by Zhu et al.^16^. Briefly, algal cells were collected by brief centrifugation. After cell lysis and centrifugation, protein concentration was quantified using the BCA method. The reaction system was constructed according to the manufacturer’s instructions, and UGPase activity was measured by monitoring absorbance changes at 30 min intervals at 340 nm using a UV-3310 spectrophotometer (Hitachi). One unit of UGPase activity was defined as 1 nmol of NADPH generated by per mg of protein per minute.

### Analysis of growth curve and photosynthetic performance

To monitor the growth of WT and transgenic strains, growth curves were constructed by measuring cell density using a hemocytometer under a light microscope every 2 days. The specific growth rate (μ, d^-1^) was determined from measurements of cell density using the following formula: (lnN_t_-lnN_0_)/(t_1_-t_0_), where N_t_ and N_0_ are cell density at time t_1_ and t_0_, respectively^53^. To analyze the effects of UGP1 and UGP2 on the photosynthetic performance of *P. tricornutum*, chlorophyll fluorescence parameters of photosystem II (PSII), such as the maximum quantum yield of PSII (*Fv*/*Fm*), the effective quantum yield (YII), and the electron transport rate (ETR) were monitored using a PAM fluorometer (Water-PAM, Walz, Germany) every 2 days during cultivation for WT and transgenic strains.

### Measurement of cellular metabolites

The algal cells were collected by centrifugation and freeze-dried overnight using an ALPHA 1-4 LD freeze dryer (Christ, Osterod, Germany) to obtain algal powder. Chrl was extracted from algal powder according to the method reported by Granum and Myklestad^54^. The extracted Chrl was quantified using the phenol-sulfuric acid method described previously by Dubois et al.^55^. Briefly, 5 mL H_2_SO_4_ (0.05 mol L^-1^) was added to 5 mg algal powder, and then extracted at 60 °C for 1 h. After repeated extraction for 3 times, all extracts were filtered through 0.22 μm filter membrane. 1 mL of Chrl extract was mixed quickly with 1 mL of 5% phenol solution (w/v), and then 5 mL of concentrated sulfuric acid was added. The mixture was mixed thoroughly and incubated at room temperature for 30 min. The signal of Chrl could be measured at 490 nm. Chrl productivity was calculated as follows:

$${{{\rm{Chrl}}}}\,{{{\rm{productivity}}}}({{{{{\rm{mg}}}}}}{{{{{{\rm{L}}}}}}}^{-1}{{{{{{\rm{day}}}}}}}^{-1})={{{{{{\rm{W}}}}}}}_{{{{{{\rm{A}}}}}}}\times {{{{{{\rm{C}}}}}}}_{{{{{{\rm{Chrl}}}}}}}/{{{{{\rm{V}}}}}}/{{{{{\rm{T}}}}}}$$

where W_A_ is the biomass (mg), C_Chrl_ is the Chrl content (%), V is the culture volume (L), and T is the culture time (day).

Soluble proteins were extracted using a plant protein extraction kit (Solarbio), and protein concentration was measured using the BCA method (Solarbio). In brief, 10 mg of algal powder was ground in liquid nitrogen, and 3 mL of lysis solution was added for 30 min lysis at low temperature. Then, 20 μL lysis solution was mixed with 200 μL BCA working solution and reacted at 37 °C for 30 min. Soluble protein concentration was calculated by monitoring absorbance changes at 562 nm.

Total lipids were extracted from the freeze-dried algal biomass using a chloroform-methanol protocol according to the method described by Zhu et al.^16^., and total lipid content was measured by gravimetric means. Briefly, 30 mg of algal powder was added to 3 mL of chloroform: methanol (2:1, v/v) and mixed by vortexing for 15 min. The lower layer (precipitate) was repeatedly extracted twice after centrifugation. Then, 2 mL of 0.9% NaCl was added to all extracts and reacted at room temperature for 15 min. Finally, the lower solution was transferred to glass tubes, and the total lipid weight was weighed after the chloroform evaporated. Lipid productivity was calculated as follows:

$${{{{{\rm{Lipid\,productivity}}}}}}({{{{{\rm{mg}}}}}}{{{{{{\rm{L}}}}}}}^{-1}{{{{{{\rm{day}}}}}}}^{-1})={{{{{{\rm{W}}}}}}}_{{{{{{\rm{A}}}}}}}\times {{{{{{\rm{C}}}}}}}_{{{{{{\rm{Lipid}}}}}}}/{{{{{\rm{V}}}}}}/{{{{{\rm{T}}}}}}$$

where W_A_ is the biomass (mg), C_Lipid_ is the lipid content (%), V is the culture volume (L), and T is the culture time (day).

### Expression analyses of UDP-sulfoquinovose synthase (SQD1) and sulfolipid synthase (SQD2) genes

qPCR was used to detect the effects of *UGP1* and *UGP2* expression levels on the key genes *SQD1* and *SOD2* in the SQDG biosynthesis pathway. The histone H4 gene was used as the internal control. Relative transcript abundance was calculated using the 2^−ΔΔCt^ method. Primers used for qPCR were shown in Supplementary Table 1. The annealing temperature of *SQD1*, *SQD2* primers was 60 °C, and their amplification efficiencies were 102.262 and 103.617, respectively.

### Detection of ROS production and cell mortality in *UGP1* and *UGP2* overexpressing lines

ROS production was determined using 2´,7´-dichlorodihydrofluorescein diacetate (DCFH-DA, Sigma) in WT and overexpression lines according to the method described by Zhao et al.^56^. In brief, DCFH-DA was added to 1 mL of *P. tricornutum* cell solution to a final concentration of 5 μM and the reaction was carried out for 45 min at room temperature in the dark. Samples were centrifuged, washed twice with ddH_2_O, and resuspended in 1 mL culture medium to a cell density of 10^7^ cells mL^-1^. Finally, the fluorescence level of each algal cell was detected by flow cytometry (EPICS XL, Beckman Coulter, High Wycombe, UK). ROS production was determined by calculating the percentage of DCF-positive cells in the total number of microalgal cells measured by flow cytometry at an excitation wavelength of 488 nm and an emission wavelength of 520 nm. CytExpert 2.3 was used for the collection and analysis of flow cytometry data. Gating strategy was shown in Supplementary Fig. 5.

To calculate the cell mortality rate of WT and overexpression lines during culture, dead cells were identified using SYTOX Green (1 µM, Invitrogen) as an indicator^56,57^. In brief, SYTOX Green was added to 1 mL of *P. tricornutum* cell solution to a final concentration of 1 μM and the reaction was carried out for 45 min at room temperature in the dark. Samples were centrifuged, washed twice with ddH_2_O, and resuspended in 1 mL culture medium to a cell density of 10^7^ cells mL^−1^. Finally, the fluorescence level of each algal cell was detected by flow cytometry. The cell death rates were determined by calculating the percentage of SYTOX-positive cells in the total number of microalgal cells measured using flow cytometry at an excitation wavelength of 488 nm and an emission wavelength of 520 nm. CytExpert 2.3 was used for the collection and analysis of flow cytometry data. Gating strategy was shown in Supplementary Fig. 5.

### RNA-seq analysis

WT and overexpression strains cultured for 6 days were used for RNA-seq. Total RNA was extracted using the Trizol reagent kit (Invitrogen) according to the manufacturer’s protocol, and RNA quality was assessed. Qualified RNA (RIN value > 7) was purified using oligo (dT) magnetic beads (Illumina) and used to synthesize cDNA. A sequencing library was constructed using synthetic cDNA and sequenced on the Illumina Novaseq 6000 platform at Gene Denovo Biotechnology Co., Ltd (Guangzhou, China). For data analysis, raw data were filtered for data quality assurance. The clean data were mapped to the reference genome (NCBI: GCA_000150955.2 ASM15095v2, *P. tricornutum*). Gene abundance was quantified using StringTie v1.3.1 and RSEM software. The DESeq2 software was used to detect differentially expressed genes (DEGs) between the WT and overexpression lines. Genes with a false discovery rate (FDR) < 0.05 and |log_2_fold change | ≥ 1 were considered as DEGs^58^.

### qPCR validation

To validate the accuracy of transcriptome results, qPCR analysis was performed on the selected 15 differentially expressed genes using the Applied Biosystems QuantStudio5 Real-Time PCR System. The histone H4 gene was used as the internal control. Relative transcript abundance was calculated using the 2^−ΔΔCt^ method. The primer sequences used in this section were shown in Supplementary Table 1.

### Statistics and reproducibility

*n* = 3 biologically independent samples. All experiments were performed in triplicate. The data were shown as the mean ± standard deviation (SD). The mean and SD for all data were calculated using Origin 9. GraphPad Prism 9 and Origin 9 were used to plot figures. One-way ANOVA was performed using SPSS 26.0. One-way ANOVA was used to determine the statistical significance of differences between treatments, and a *p*-value < 0.05 was considered statistically significant. The raw data used for data analysis were in Supplementary Data, and the exact *p*-values were available in Supplementary Data 22.

### Reporting summary

Further information on research design is available in the Nature Portfolio Reporting Summary linked to this article.

## Supplementary information

Supplementary Information

Description of Additional Supplementary Files

Supplementary Data 1-22

Reporting Summary

## Data Availability

The source data behind the graphs in the paper were available in Supplementary Data. Uncropped and unedited blot/gel images for Fig. 3a and c were presented in Supplementary Fig. 6 and 7. RNA-seq raw sequence data have been deposited in the Genome Sequence Archive (GSA) database in National Genomics Data Center under the accession number (GSA: CRA011416). All constructed plasmids used pPha-T1 as backbone, and plasmid pPha-T1 was submitted to GenBank with ID number AF219942.1^50^. All other data are available from the corresponding author upon reasonable request.

## References

[1] Foong CP (2020). A marine photosynthetic microbial cell factory as a platform for spider silk production. Commun. Biol..

[2] Hu Q (2008). Microalgal triacylglycerols as feedstocks for biofuel production: perspectives and advances. Plant J..

[3] Jia J (2015). Molecular mechanisms for photosynthetic carbon partitioning into storage neutral lipids in *Nannochloropsis oceanica* under nitrogen-depletion condition. Algal. Res..

[4] Ambati RR, Gogisetty D, Aswathanarayana RG, Ravi S, Yuepeng S (2018). Industrial potential of carotenoid pigments from microalgae: current trends and future prospects. Crit. Rev. Food Sci. Nutr..

[5] Yang R, Wei D, Xie J (2020). Diatoms as cell factories for high-value products: chrysolaminarin, eicosapentaenoic acid, and fucoxanthin. Crit. Rev. Biotechnol..

[6] Butler T, Kapoore RV, Vaidyanathan S (2020). *Phaeodactylum tricornutum*: a diatom cell factory. Trends Biotechnol..

[7] Sun H (2020). Harnessing C/N balance of *Chromochloris zofingiensis* to overcome the potential conflict in microalgal production. Commun. Biol..

[8] Choi BY (2021). The Chlamydomonas bZIP transcription factor BLZ8 confers oxidative stress tolerance by inducing the carbon-concentrating mechanism. Plant Cell.

[9] Ding XT (2021). Expression of the Vitreoscilla hemoglobin gene in *Nannochloropsis oceanica* regulates intracellular oxygen balance under high-light. J. Photochem. Photobiol. B Biol..

[10] Ohlrogge JB, Browse JG (1995). Lipid biosynthesis. Plant Cell.

[11] Michel G, Tonon T, Scornet D, Cock JM, Kloareg B (2010). Central and storage carbon metabolism of the brown alga *Ectocarpus siliculosus*: insights into the origin and evolution of storage carbohydrates in Eukaryotes. N. Phytologist.

[12] Heydarizadeh P (2019). Carbon orientation in the diatom *Phaeodactylum tricornutum*: the effects of carbon limitation and photon flux density. Front. Plant Sci..

[13] Xia S (2014). Preliminary characterization, antioxidant properties and production of chrysolaminarin from marine diatom *Odontella aurita*. Mar. Drugs.

[14] Yang YF (2019). Overproduction of bioactive algal chrysolaminarin by the critical carbon flux regulator phosphoglucomutase. Biotechnol. J..

[15] Caballero MA (2016). Quantification of chrysolaminarin from the model diatom *Phaeodactylum tricornutum*. Algal. Research.

[16] Zhu BH (2016). Silencing UDP-glucose pyrophosphorylase gene in *Phaeodactylum tricornutum* affects carbon allocation. N. Biotechnol..

[17] Wu SC (2019). Elevated CO_2_ improves both lipid accumulation and growth rate in the glucose-6-phosphate dehydrogenase engineered *Phaeodactylum tricornutum*. Microb. Cell Factories.

[18] Valenzuela J (2013). Nutrient resupplementation arrests bio-oil accumulation in *Phaeodactylum tricornutum*. Appl. Microbiol. Biotechnol..

[19] Yang ZK (2013). Molecular and cellular mechanisms of neutral lipid accumulation in diatom following nitrogen deprivation. Biotechnol. Biofuels.

[20] Yodsuwan N, Sawayama S, Sirisansaneeyakul S (2017). Effect of nitrogen concentration on growth, lipid production and fatty acid profiles of the marine diatom *Phaeodactylum tricornutum*. Agriculture Nat. Resour..

[21] Xue J, Niu YF, Huang T, Yang WD, Li HY (2015). Genetic improvement of the microalga *Phaeodactylum tricornutum* for boosting neutral lipid accumulation. Metab. Eng..

[22] Hao X (2021). Multiplexed CRISPR/Cas9 editing of the long-chain acyl-CoA synthetase family in the diatom *Phaeodactylum tricornutum* reveals that mitochondrial ptACSL3 is involved in the synthesis of storage lipids. N. Phytologist.

[23] Daboussi F (2014). Genome engineering empowers the diatom *Phaeodactylum tricornutum* for biotechnology. Nat. Commun..

[24] Meng M, Wilczynska M, Kleczkowski LA (2008). Molecular and kinetic characterization of two UDP-glucose pyrophosphorylases, products of distinct genes, from *Arabidopsis*. Biochim. et. Biophys. Acta (BBA) - Proteins Proteom..

[25] Xiao G, Zhou J, Lu X, Huang R, Zhang H (2018). Excessive UDPG resulting from the mutation of UAP1 causes programmed cell death by triggering reactive oxygen species accumulation and caspase-like activity in rice. N. Phytologist.

[26] Coleman HD, Canam T, Kang KY, Ellis DD, Mansfield SD (2007). Over-expression of UDP-glucose pyrophosphorylase in hybrid poplar affects carbon allocation. J. Exp. Bot..

[27] Wang QH (2011). Identification of a UDP-glucose pyrophosphorylase from cotton (*Gossypium hirsutum* L.) involved in cellulose biosynthesis in *Arabidopsis thaliana*. Plant Cell Rep..

[28] Huang W, Rio BC, Kroth PG (2016). Diatom vacuolar 1,6-beta-transglycosylases can functionally complement the respective yeast mutants. J. Eukaryot. Microbiol..

[29] Kroth PG (2008). A model for carbohydrate metabolism in the diatom *Phaeodactylum tricornutum* deduced from comparative whole genome analysis. Plos One.

[30] Okazaki Y (2009). A chloroplastic UDP-glucose pyrophosphorylase from *Arabidopsis* is the committed enzyme for the first step of sulfolipid biosynthesis. Plant Cell.

[31] Sun C (2011). *RLIN1*, encoding a putative coproporphyrinogen III oxidase, is involved in lesion initiation in rice. J. Genet. Genom..

[32] Ward NP, Kang YP, Falzone A, Boyle TA, Denicola GM (2020). Nicotinamide nucleotide transhydrogenase regulates mitochondrial metabolism in NSCLC through maintenance of Fe-S protein function. J. Exp. Med..

[33] Bidle KD (2016). Programmed cell death in unicellular phytoplankton. Curr. Biol..

[34] Bucher M, Brändle R, Kuhlemeier C (1994). Ethanolic fermentation in transgenic tobacco expressing Zymomonas mobilis pyruvate decarboxylase. The. EMBO J..

[35] Lu Y, Wu YR, Han B (2005). Anaerobic induction of isocitrate lyase and malate synthase in submerged rice seedlings indicates the important metabolic role of the glyoxylate cycle. Acta Biochim. et. Biophys. Sin..

[36] Li Y (2010). Chlamydomonas starchless mutant defective in ADP-glucose pyrophosphorylase hyper-accumulates triacylglycerol. Metab. Eng..

[37] Sowokinos JR, Vigdorovich V, Abrahamsen A (2004). Molecular cloning and sequence variation of UDP-glucose pyrophosphorylase cDNAs from potatoes sensitive and resistant to cold sweetening. J. Plant Physiol..

[38] Kleczkowski LA, Geisler M, Fitzek E, Wilczynska M (2011). A common structural blueprint for plant UDP-sugar-producing pyrophosphorylases. Biochem. J..

[39] Katsube T, Kazuta Y, Tanizawa K, Fukui T (1991). Expression in Escherichia coli of UDP-glucose pyrophosphorylase cDNA from potato tuber and functional assessment of the five lysyl residues located at the substrate-binding site. Biochemistry.

[40] Geisler M, Wilczynska M, Karpinski S, Kleczkowski LA (2004). Toward a blueprint for UDP-glucose pyrophosphorylase structure/function properties: homology-modeling analyses. Plant Mol. Biol..

[41] Kleczkowski LA, Kunz S, Wilczynska M (2010). Mechanisms of UDP-glucose synthesis in plants. Crit. Rev. Plant Sci..

[42] Volkman JK, Brown MR, Dunstan GA, Jeffrey SW (1993). The biochemical composition of marine microalgae from the class Eustigmatophyceae1. J. Phycol..

[43] Deng X, Cai J, Li Y, Fei X (2014). Expression and knockdown of the PEPC1 gene affect carbon flux in the biosynthesis of triacylglycerols by the green alga *Chlamydomonas reinhardtii*. Biotechnol. Lett..

[44] Hildebrand M, Manandhar-Shrestha K, Abbriano R (2017). Effects of chrysolaminarin synthase knockdown in the diatom *Thalassiosira pseudonana*: Implications of reduced carbohydrate storage relative to green algae. Algal Res..

[45] Ding Q, Diao W, Gao C, Chen X, Liu L (2020). Microbial cell engineering to improve cellular synthetic capacity. Biotechnol. Adv..

[46] Fang Y (2020). Rebalancing microbial carbon distribution for L-threonine maximization using a thermal switch system. Metab. Eng..

[47] Radakovits R, Eduafo PM, Posewitz MC (2011). Genetic engineering of fatty acid chain length in *Phaeodactylum tricornutum*. Metab. Eng..

[48] Marchler-Bauer A (2015). CDD: NCBI’s conserved domain database. Nucleic Acids Res..

[49] Emanuelsson O, Brunak S, von Heijne G, Nielsen H (2007). Locating proteins in the cell using TargetP, SignalP and related tools. Nat. Protoc..

[50] Zaslavskaia LA, Lippmeier JC, Kroth PG, Grossman AR, Apt KE (2000). Transformation of the diatom *Phaeodactylum tricornutum* (Bacillariophyceae) with a variety of selectable marker and reporter genes. J. Phycol..

[51] Siaut M (2007). Molecular toolbox for studying diatom biology in *Phaeodactylum tricornutum*. Gene.

[52] Livak KJ, Schmittgen TD (2001). Analysis of relative gene expression data using real-time quantitative PCR and the 2^−ΔΔCt^ method. Methods.

[53] Lim SL, Chu WL, Phang SM (2010). Use of *Chlorella vulgaris* for bioremediation of textile wastewater. Bioresour. Technol..

[54] Granum E, Myklestad SM (2002). A simple combined method for determination of b-1,3-glucan and cell wall polysaccharides in diatoms. Hydrobiologia.

[55] Dubois M, Gilles KA, Hamilton JK, Rebers P, Smith F (1956). Colorimetric method for determination of sugars and related substances. Anal. Chem..

[56] Zhao Y, Tang X, Qu F, Lv M, Zhao Y (2020). ROS-mediated programmed cell death (PCD) of *Thalassiosira pseudonana* under the stress of BDE-47. Environ. Pollut..

[57] Peperzak L, Brussaard CPD (2011). Flow cytometric applicability of fluorescent vitality probes on phytoplankton. J. Phycol..

[58] Rajkumar AP (2015). Experimental validation of methods for differential gene expression analysis and sample pooling in RNA-seq. BMC Genom..

