# Supplementary material for: UDP-glucose pyrophosphorylase as a target for regulating carbon flux distribution and antioxidant capacity in Phaeodactylum tricornutum
Source: Commun Biol. 2023 Jul 19;6:750. doi: 10.1038/s42003-023-05096-3 (PMC10356853; doi:10.1038/s42003-023-05096-3)
Supplement: Supplementary file 1 — Supplementary Information [file 42003_2023_5096_MOESM1_ESM.pdf]

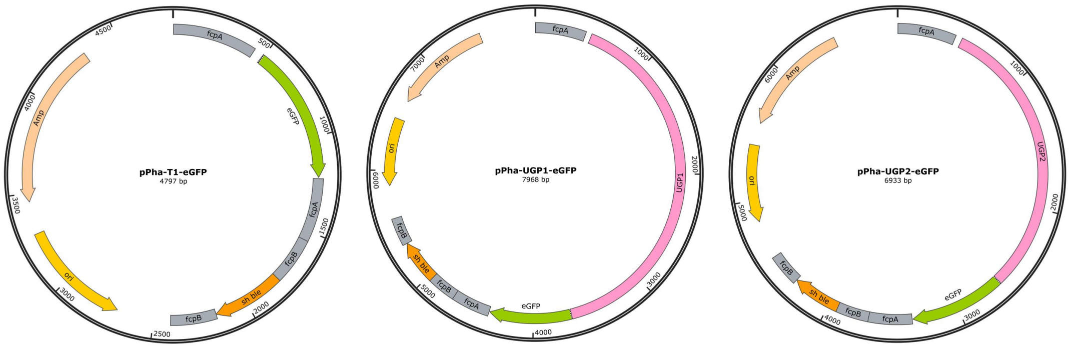

Supplementary Fig. 1. Maps of vectors for studying subcellular localization. pPha-T1-eGFP: vector for transformation of eGFP alone; pPha-UGP1-eGFP: vector for transformation of UGP1 fused to eGFP; pPha-UGP2-eGFP: vector for transformation of UGP2 fused to eGFP.

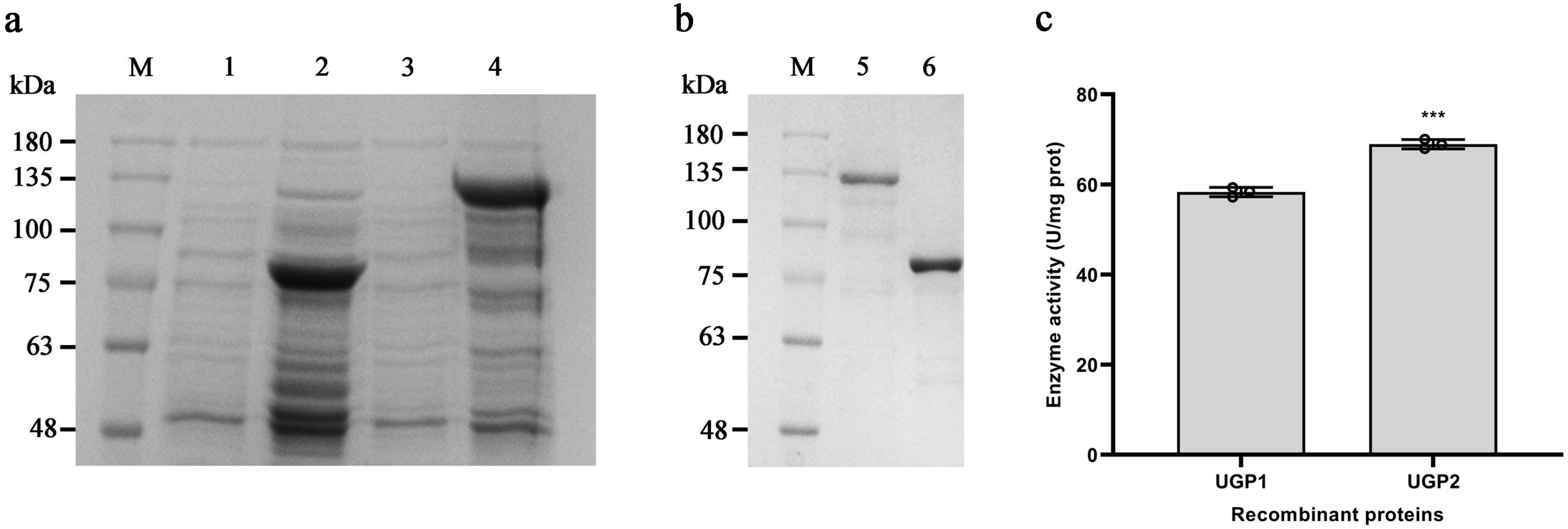

Supplementary Fig. 2 Purification and enzymatic activity analysis of recombinant UGP1 and UGP2 proteins. a SDS-PAGE analysis of IPTG-stimulated *E. coli* crude lysate. Lanes 1 and 3 show *E. coli* crude lysate containing the pEASY-Blunt E2 vector (empty vector control). Lanes 2 and 4 show *E. coli* crude lysate containing pEASY-UGP2 and pEASY-UGP1 recombinant vectors, respectively. M, protein marker. **b** SDS-PAGE analysis of purified recombinant proteins. Lanes 5 and 6 show the purified UGP1 and UGP2 proteins, respectively. M, protein marker. **c** Enzyme activity of UGP1 and UGP2 recombinant proteins. UGP1 and UGP2, recombinant UDP-glucose pyrophosphorylase. n = 3 biologically independent samples. The data were shown as the mean ± standard deviation (SD). Two proteins enzymatic activities were compared using independent samples T-test, ***$p < 0.001$.

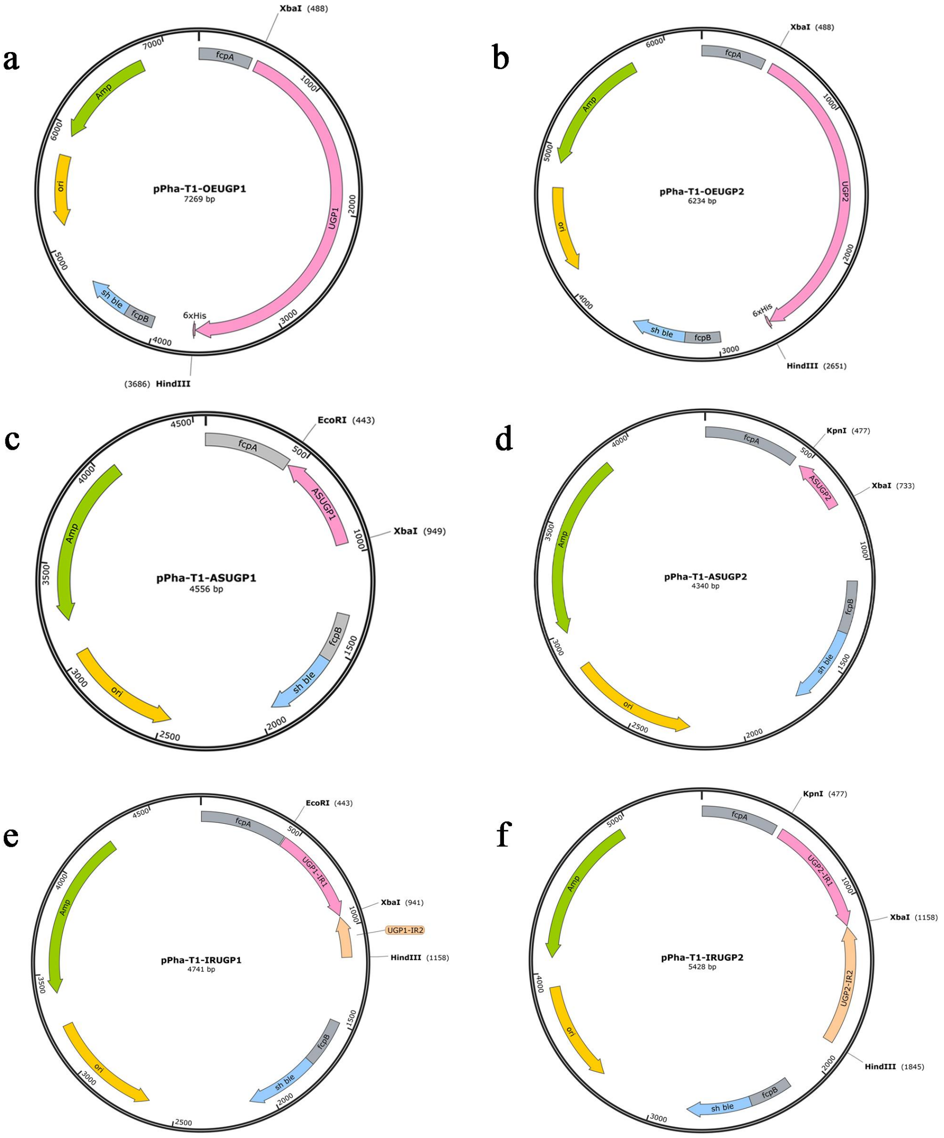

Supplementary Fig. 3 Maps of overexpression and silencing vectors. a pPha-T1-OEUGP1 overexpression vector. **b** pPha-T1-OEUGP2 overexpression vector. **c** pPha-T1-ASUGP1 silencing vector. **d** pPha-T1-ASUGP2 silencing vector. **e** pPha-T1-IRUGP1 silencing vector. **f** pPha-T1-IRUGP2 silencing vector.

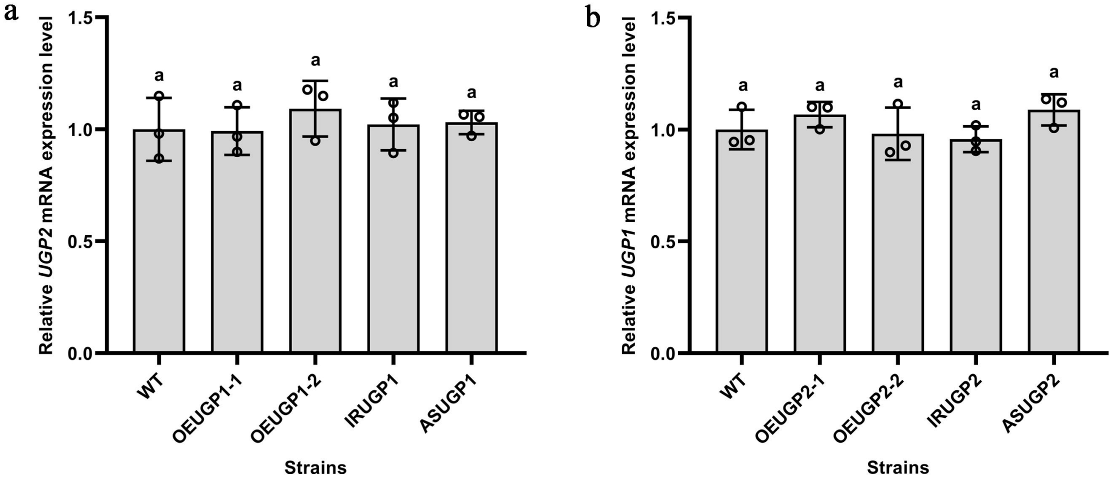

Supplementary Fig. 4 a The expression level of *UGP2* in *UGP1*-overexpressing and silenced strains. **b** The expression level of *UGP1* in *UGP2*-overexpressing and silenced strains. ASUGP1 and IRUGP1 represent *UGP1* silenced strains; OEUGP1-1 and OEUGP1-2 represent *UGP1*-overexpressing strains; ASUGP2 and IRUGP2 represent *UGP2* silenced strains; OEUGP2-1 and OEUGP2-2 represent *UGP2*-overexpressing strains. n = 3 biologically independent samples. The data were shown as the mean ± standard deviation (SD). Differences among groups were determined by one-way ANOVA and Tukey's test. Values with same letters (a) indicate no significant difference between them (p > 0.05).

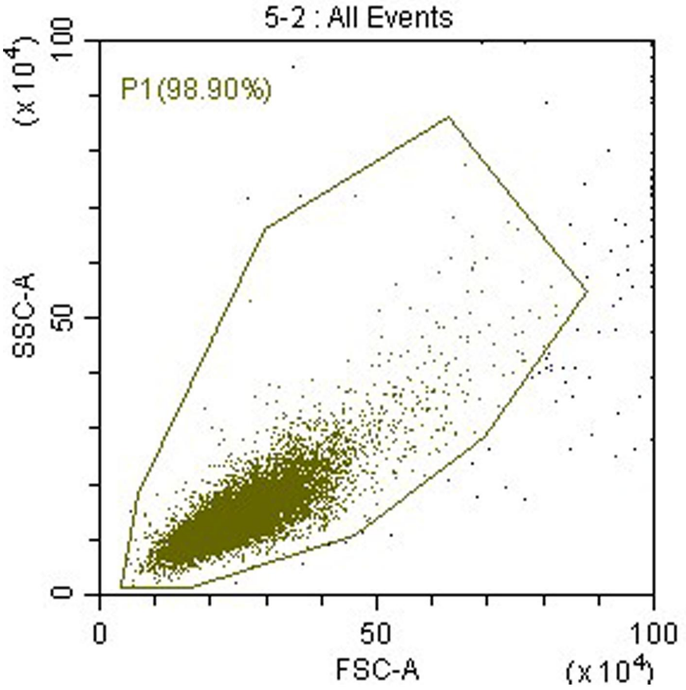

Supplementary Fig. 5 The gating strategy according to FSC/SSC.

Fig. 3a

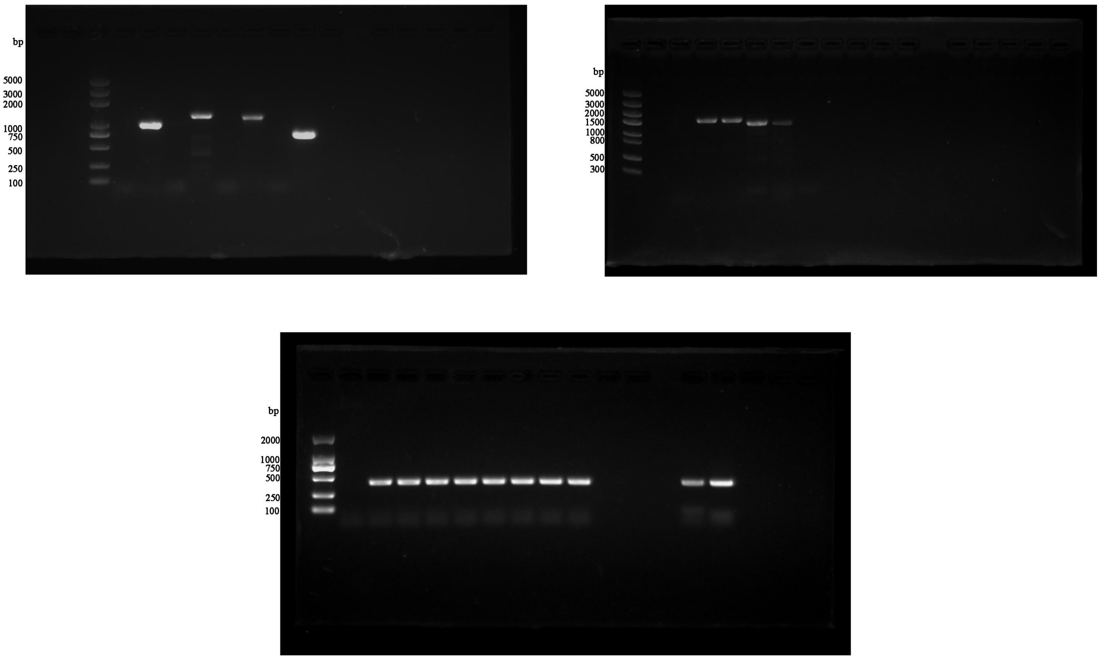

Supplementary Fig. 6 Original uncropped images for Fig. 3a.

Fig. 3c

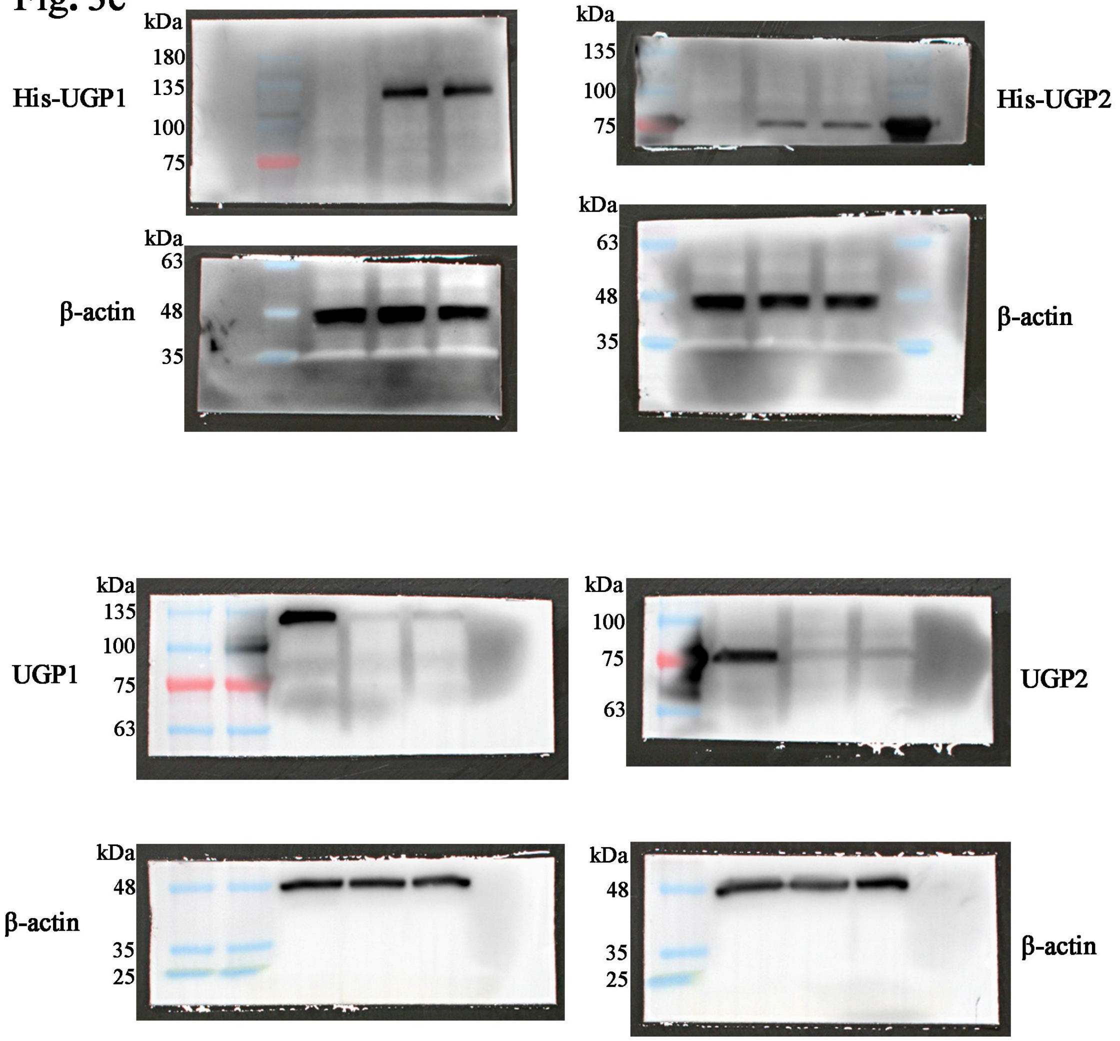

Supplementary Fig. 7 Original uncropped images for Fig. 3c.

Supplementary Table 1. List of primers used in this study.

Primer name	Primer sequence (5'→3')	Purpose
PtUGP1-f	GCTCTAGAATGCCTTCTTTCGATCCCATTCGTG	Gene cloning and overexpression vector construction
PtUGP1-r	CCCAAGCTTTTAATGATGATGATGATGATGCGTAATTACGGTTGGTTC	Gene cloning and overexpression vector construction
PtUGP2-f	GCTCTAGAATGAGACTAGCCATTGCTGTTTCCTTTTTG	Gene cloning and overexpression vector construction
PtUGP2-r	CCCAAGCTTTTAATGATGATGATGATGATGCTCTGCCGCCTTTTGCACATTT	Gene cloning and overexpression vector construction
YH-UGP1-f	ATGCCTTCTTTCGATCCCATTCGTGC	Prokaryotic expression vector construction
YH-UGP1-r	CGTAATTACGGTTGGTTC	Prokaryotic expression vector construction
YH-UGP2-f	TTCGGAACCCCGGCCAAAAAC	Prokaryotic expression vector construction
YH-UGP2-r	CTCTGCCGCCTTTTGCACATTT	Prokaryotic expression vector construction
UGP1-IR1-f	CGGAATTCTGCTATGTGCGCTGAA	Silencing vector construction
UGP1-IR1-r	CCCTCTAGACCAAACCCTTGATAGTC	Silencing vector construction
UGP1-IR2-f	GGAAGCTTGCTATGTGCGCTGAA	Silencing vector construction
UGP1-IR2-r	CCGTCTAGATAGCGGTTTCCAACT	Silencing vector construction
UGP1-AS-f	CGTCTAGAGGGAATCTGCTATGTGC	Silencing vector construction
UGP1-AS-r	CGGAATTCGACCAAACCCTTGATAGTC	Silencing vector construction
UGP2-IR1-f	GGAAGCTTACTACACGACTCCGACAAAC	Silencing vector construction
UGP2-IR1-r	CCCTCTAGAGTCCTGCCACTGATCCAC	Silencing vector construction
UGP2-IR2-f	CGGGGTACCACTACACGACTCCGACAAAC	Silencing vector construction
UGP2-IR2-r	CCCTCTAGACCTGCCACTGATCCACAA	Silencing vector construction
UGP2-AS-f	GCTCTAGAGCCCTGGACGACAAAGAT	Silencing vector construction
UGP2-AS-r	CGGGGTACCATGGCACCCGCTGCTTCA	Silencing vector construction
Sh ble-f	CACGGTTGCCAGATGTCAAGATGGCCAAG	Molecular identification
Sh ble-r	GGTTCAGTCCTGCTCCTCGGCCACGAAGTG	Molecular identification
OEUGP1-f	CCGAATACACTCCGTTTA	Molecular identification
OEUGP1-r	GGTTCAGTCCTGCTCCT	Molecular identification
OEUGP2-f	TTCCAGTCACGATGGCGGACGATTT	Molecular identification
OEUGP2-r	GGTTCAGTCCTGCTCCTCGGCCA	Molecular identification
IRUGP1-f	CTCAATCCAGCCTGCAACG	Molecular identification
IRUGP1-r	GACACGACCTCCGACCACT	Molecular identification
ASUGP1-f	GAGCAGATCGCTGCACTTT	Molecular identification
ASUGP1-r	GAACGGCACTGGTCAACTT	Molecular identification
IRUGP2-f	GGAAGCTTACTACACGACTCCGACAAAC	Molecular identification

IRUGP2-r	CCCTCTAGAGTCCTGCCACTGATCCAC	Molecular identification
ASUGP2-f	ATGGCACCCGCTGCTTCA	Molecular identification
ASUGP2-r	ACACGACCTCCGACCACTCG	Molecular identification
RT-UGP1-f	ATTGCGGGACAAAAGCCTGGT	qPCR
RT-UGP1-r	CGCCGTTAGCAACACCCATCT	qPCR
RT-UGP2-f	CCAAAAGGAAATGGGCGCCG	qPCR
RT-UGP2-r	AGCCAGCTTGCGTTGGTTCT	qPCR
RT-H4-f	GTGGTAAAGGAGGCAAGGGTC	qPCR
RT-H4-r	GGCGTGCTCGGTATAGGTGA	qPCR
SQD1-f	CAGAGGTCGACGCCCATATCAT	qPCR
SQD1-r	CAAGCACTTGGTGGCATGGTAG	qPCR
SQD2-f	CAACGGGATTCCTCGAGTGGAC	qPCR
SQD2-r	GTTGGGCATTCGTTCCAGCATG	qPCR
NNT-f	GACTTGTGTTCTAGGTTTCGCTG	qPCR
NNT-r	GATGATAGCCGAGAATTGATGCG	qPCR
CPOX-f	TGGAGGTGGCACAGATATAACTC	qPCR
CPOX-r	GATCAAAAAGTACTCATCGGCCC	qPCR
MCA-f	GAGTTTCACATGATCTCGGGTTC	qPCR
MCA-r	TTGTAGAGGACCTGTAACAAGGC	qPCR
CDPK-1-f	TCAGTCTCCTTTCCATCCTTACG	qPCR
CDPK-1-r	GATAGTCGGCATCGTACCTTTTG	qPCR
CDPK-2-f	GGTCTTGCTTTATTCTGTGTCCC	qPCR
CDPK-2-r	CTTTTCATGGGTAAGTGCTCCTG	qPCR
GAPDH-f	GATCCCTTCATTCCCGTCGAATA	qPCR
GAPDH-r	ACCTTGATTTCGTTCTCACCCA	qPCR
MetH-f	GAATATCGTCGCTGTTGTCCTTG	qPCR
MetH-r	ATCCAGAGAGACCAATCACATCG	qPCR
GST-f	ACTAATTGAAGACGAGATGGGGG	qPCR
GST-r	AGGGACCTTTTTGATAGTCAGGG	qPCR
Ccp1-f	CAAATGAATGGCCCCACTATTCC	qPCR
Ccp1-r	CTCTTGGTCGTTGAAACCCATAC	qPCR
P4H-1-f	CATCCTTCCTCCGTACCTACTTG	qPCR
P4H-1-r	AATCTAGTAATGTCCCCAGTGGC	qPCR
P4H-2-f	CAGGTGACCTTGTCCTGTATGAA	qPCR

P4H-2-r	CGTCGAGTTCGTCCAAATATTCC	qPCR
P4H-3-f	GGCGAGACGATTCCTTTAGATTC	qPCR
P4H-3-r	GATTTGGTTGTCGGTGCTTCTAG	qPCR
PGK-f	GATGCTGGTATTGACGATGGTTG	qPCR
PGK-r	CGTTCTTGGTGAGGGATTCAATG	qPCR
ICL-f	AATACCACTCGAGTGTACTCCTC	qPCR
ICL-r	CAAAAGACTCCAGAGTTTGTCCG	qPCR
PYC1-f	TCTTGGAAGGAGTAGTTGACACG	qPCR
PYC1-r	ACGGGTATGATAGGATCTACCGT	qPCR

Supplementary Table 2. Genes list of Fig. 7b.

Gene	Full Name	Gene ID	$\log_2$(fold change) between the pairwise comparisons	
			WT vs OEUGP1	WT vs OEUGP2
CPOX	coproporphyrinogen III oxidase	7204128	2.27	0.37
NNT	$NAD(P)^+$ transhydrogenase	7204049	1.08	-0.38
MCA	metacaspase	7203298	-1.05	0.24
CDPK	calcium dependent protein kinase	7195076	-1.64	0.52
CDPK	calcium dependent protein kinase	7197935	-1.01	-0.15
GAPDH	glyceraldehyde-3-phosphate dehydrogenase	7197206	-1.84	0.97
MetH	methionine synthase	7198319	-1.17	-0.29
GST	glutathione S-transferase	7200010	-1.65	0.80
Ccp1	cytochrome c peroxidase	7204592	-1.01	0.11
P4H	prolyl 4-hydroxylase	7204536	2.17	0.31
P4H	prolyl 4-hydroxylase	7198746	1.13	-0.05
P4H	prolyl 4-hydroxylase	7199826	1.56	0.20
PGK	phosphoglycerate kinase	7203354	1.67	-0.82
ICL	isocitrate lyase	7202968	1.04	-0.50
PYC1	pyruvate carboxylase	7198165	1.29	-0.32

Supplementary Table 3**.** Pearson correlation analysis of *UGP2* transcript abundance with *SQD1* and *SQD2* transcription levels.

Genes transcription levels	Pearson correlation coefficient	
	SQD1	SQD2
UGP2	0.972*	0.989*

* Indicates statistical significance, $p < 0.05$
