# Supplementary material for: UDP-glucose pyrophosphorylase as a target for regulating carbon flux distribution and antioxidant capacity in Phaeodactylum tricornutum
Source: Commun Biol. 2023 Jul 19;6:750. doi: 10.1038/s42003-023-05096-3 (PMC10356853; doi:10.1038/s42003-023-05096-3)
Supplement: Supplementary file 2 — Description of Additional Supplementary Files [file 42003_2023_5096_MOESM2_ESM.pdf]

# Description of Additional Supplementary Files

**File name:** Supplementary Data 1-21

**Description:** The source data behind the graphs in the paper.

**File name:** Supplementary Data 22

**Description:** The exact p-values of data analysis.
