# Supplementary material for: UDP-glucose pyrophosphorylase as a target for regulating carbon flux distribution and antioxidant capacity in Phaeodactylum tricornutum
Source: Commun Biol. 2023 Jul 19;6:750. doi: 10.1038/s42003-023-05096-3 (PMC10356853; doi:10.1038/s42003-023-05096-3)
Supplement: Supplementary file 4 — Reporting Summary [file 42003_2023_5096_MOESM4_ESM.pdf]

nature portfolio

Corresponding author(s): DBPR COMMSBIO-22-2293A

Last updated by author(s): Jun 20, 2023

Reporting Summary

Nature Portfolio wishes to improve the reproducibility of the work that we publish. This form provides structure for consistency and transparency in reporting. For further information on Nature Portfolio policies, see our Editorial Policies and the Editorial Policy Checklist.

Statistics

For all statistical analyses, confirm that the following items are present in the figure legend, table legend, main text, or Methods section.

n/a	Confirmed	
☐	☒	The exact sample size (*n*) for each experimental group/condition, given as a discrete number and unit of measurement
☐	☒	A statement on whether measurements were taken from distinct samples or whether the same sample was measured repeatedly
☐	☒	The statistical test(s) used AND whether they are one- or two-sided<br>*Only common tests should be described solely by name; describe more complex techniques in the Methods section.*
☒	☐	A description of all covariates tested
☒	☐	A description of any assumptions or corrections, such as tests of normality and adjustment for multiple comparisons
☐	☒	A full description of the statistical parameters including central tendency (e.g. means) or other basic estimates (e.g. regression coefficient) AND variation (e.g. standard deviation) or associated estimates of uncertainty (e.g. confidence intervals)
☐	☒	For null hypothesis testing, the test statistic (e.g. *F*, *t*, *r*) with confidence intervals, effect sizes, degrees of freedom and *P* value noted<br>*Give P values as exact values whenever suitable.*
☒	☐	For Bayesian analysis, information on the choice of priors and Markov chain Monte Carlo settings
☒	☐	For hierarchical and complex designs, identification of the appropriate level for tests and full reporting of outcomes
☐	☒	Estimates of effect sizes (e.g. Cohen's *d*, Pearson's *r*), indicating how they were calculated

Our web collection on statistics for biologists contains articles on many of the points above.

Software and code

Policy information about availability of computer code

Data collection	no software was used
Data analysis	GraphPad Prism 9, Origin 9 and SPSS 26.0 were used for data analysis

For manuscripts utilizing custom algorithms or software that are central to the research but not yet described in published literature, software must be made available to editors and reviewers. We strongly encourage code deposition in a community repository (e.g. GitHub). See the Nature Portfolio guidelines for submitting code & software for further information.

Data

Policy information about availability of data

All manuscripts must include a data availability statement. This statement should provide the following information, where applicable:

- Accession codes, unique identifiers, or web links for publicly available datasets
- A description of any restrictions on data availability
- For clinical datasets or third party data, please ensure that the statement adheres to our policy

The source data behind the graphs in the paper are available in Supplementary Data. Uncropped and unedited blot/gel images for Fig. 3a and c are presented in Supplementary Fig. 6 and 7. RNA-seq raw sequence data have been deposited in the Genome Sequence Archive (GSA) database in National Genomics Data Center under the accession number (GSA: CRA011416) that are publicly accessible at https://ngdc.cncb.ac.cn/gsa. All other data are available from the corresponding author upon reasonable request.

Human research participants

Policy information about studies involving human research participants and Sex and Gender in Research.

Reporting on sex and gender	Not apply
Population characteristics	Not apply
Recruitment	Not apply
Ethics oversight	Not apply

Note that full information on the approval of the study protocol must also be provided in the manuscript.

Field-specific reporting

Please select the one below that is the best fit for your research. If you are not sure, read the appropriate sections before making your selection.

☒ Life sciences ☐ Behavioural & social sciences ☐ Ecological, evolutionary & environmental sciences

For a reference copy of the document with all sections, see nature.com/documents/nr-reporting-summary-flat.pdf

Life sciences study design

All studies must disclose on these points even when the disclosure is negative.

Sample size	No statistical methods were used to predetermine sample size. The sample sizes for experiments were also determined based on the reproducibility of experiments, which were usually 3.
Data exclusions	No data were excluded from the analysises.
Replication	Each experiment was repeated at least three times in independent experiments.
Randomization	The samples allocated into experimental groups were random.
Blinding	The investigators were not blinded to group allocation, because no grouping was needed for this study.

Reporting for specific materials, systems and methods

We require information from authors about some types of materials, experimental systems and methods used in many studies. Here, indicate whether each material, system or method listed is relevant to your study. If you are not sure if a list item applies to your research, read the appropriate section before selecting a response.

Materials & experimental systems

n/a	Involved in the study	
☐	☒	Antibodies
☒	☐	Eukaryotic cell lines
☒	☐	Palaeontology and archaeology
☒	☐	Animals and other organisms
☒	☐	Clinical data
☒	☐	Dual use research of concern

Methods

n/a	Involved in the study	
☒	☐	ChIP-seq
☐	☒	Flow cytometry
☒	☐	MRI-based neuroimaging

Antibodies

Antibodies used	Four antibodies including anti-UGP1, anti-UGP2, anti-His and anti-β-actin were used in the study. Anti-UGP1 and anti-UGP2 antibodies were produced by Genecreate Biological Engineering, Wuhan, China. Anti-His and anti-β-actin antibodies were purchased from Cell Signaling Technology, USA.
Validation	Anti-UGP1 and anti-UGP2 antibodies were validated by ELISA and WB. His-Tag (D3I1O) XP® Rabbit mAb and β-Actin (13E5) Rabbit mAb purchased from Cell Signaling Technology were used for western blot analysis in the experimental species of Phaeodactylum tricornutum.

Flow Cytometry

Plots

Confirm that:

☐ The axis labels state the marker and fluorochrome used (e.g. CD4-FITC).

☐ The axis scales are clearly visible. Include numbers along axes only for bottom left plot of group (a 'group' is an analysis of identical markers).

☐ All plots are contour plots with outliers or pseudocolor plots.

☒ A numerical value for number of cells or percentage (with statistics) is provided.

Methodology

Sample preparation	DCFH-DA was added to 1 mL of P. tricornutum cell solution to a final concentration of 5 μM and the reaction was carried out for 45 min at room temperature in the dark. Samples were centrifuged, washed twice with ddH2O, and resuspended in 1 mL culture medium. The fluorescence intensity was measured by flow cytometry (EPICS XL, Beckman Coulter, High Wycombe, UK) at an excitation wavelength of 488 nm and an emission wavelength of 520 nm.<br>SYTOX Green was added to 1 mL of P. tricornutum cell solution to a final concentration of 1 μM and the reaction was carried out for 45 min at room temperature in the dark. Samples were centrifuged, washed twice with ddH2O, and resuspended in 1 mL culture medium. The fluorescence intensity was measured by flow cytometry (EPICS XL, Beckman Coulter, High Wycombe, UK) at an excitation wavelength of 488 nm and an emission wavelength of 520 nm.
Instrument	flow cytometry (EPICS XL, Beckman Coulter, High Wycombe, UK)
Software	CytExpert 2.3
Cell population abundance	No flow sorting was performed.
Gating strategy	Gating strategy according to FSC and SSC.

☒ Tick this box to confirm that a figure exemplifying the gating strategy is provided in the Supplementary Information.
